# Individual bacteria in structured environments rely on phenotypic resistance to phage

**DOI:** 10.1371/journal.pbio.3001406

**Published:** 2021-10-12

**Authors:** Erin L. Attrill, Rory Claydon, Urszula Łapińska, Mario Recker, Sean Meaden, Aidan T. Brown, Edze R. Westra, Sarah V. Harding, Stefano Pagliara

**Affiliations:** 1 Living Systems Institute and Biosciences, University of Exeter, Exeter, United Kingdom; 2 SUPA, School of Physics and Astronomy, The University of Edinburgh, United Kingdom; 3 Centre for Ecology and Conservation, University of Exeter, Penryn, United Kingdom; 4 Environment and Sustainability Institute and Biosciences, University of Exeter, Penryn, United Kingdom; 5 Department of Microbiology and Immunology, University of Otago, Dunedin, New Zealand; 6 Defence Science and Technology Laboratory, Porton Down, Salisbury, United Kingdom; Max-Planck-Institut fur terrestrische Mikrobiologie, GERMANY

## Abstract

Bacteriophages represent an avenue to overcome the current antibiotic resistance crisis, but evolution of genetic resistance to phages remains a concern. In vitro, bacteria evolve genetic resistance, preventing phage adsorption or degrading phage DNA. In natural environments, evolved resistance is lower possibly because the spatial heterogeneity within biofilms, microcolonies, or wall populations favours phenotypic survival to lytic phages. However, it is also possible that the persistence of genetically sensitive bacteria is due to less efficient phage amplification in natural environments, the existence of refuges where bacteria can hide, and a reduced spread of resistant genotypes. Here, we monitor the interactions between individual planktonic bacteria in isolation in ephemeral refuges and bacteriophage by tracking the survival of individual cells. We find that in these transient spatial refuges, phenotypic resistance due to reduced expression of the phage receptor is a key determinant of bacterial survival. This survival strategy is in contrast with the emergence of genetic resistance in the absence of ephemeral refuges in well-mixed environments. Predictions generated via a mathematical modelling framework to track bacterial response to phages reveal that the presence of spatial refuges leads to fundamentally different population dynamics that should be considered in order to predict and manipulate the evolutionary and ecological dynamics of bacteria–phage interactions in naturally structured environments.

## Abstract 2

Bacteriophages represent a promising avenue to overcome the current antibiotic resistance crisis, but evolution of phage resistance remains a concern. This study shows that in the presence of spatial refuges, genetic resistance to phage is less of a problem than commonly assumed, but the persistence of genetically susceptible bacteria suggests that eradicating bacterial pathogens from structured environments may require combined phage-antibiotic therapies.

## Introduction

Viruses that infect bacteria (bacteriophages) are the most abundant entities on the planet and are critical determinants in maintaining microbial diversity within populations and communities across ecosystems [1–4]. Moreover, the emergence of antimicrobial resistance has caused a resurgence of interest in using phages in therapeutic settings [5,6], stimulating research on the arsenal of antiphage mechanisms that bacteria have evolved in order to survive [7,8]. Specifically, *Escherichia coli* and its lytic phage T4 have been extensively employed to increase our molecular understanding of phage biology [9,10], and T4 has been considered for phage therapy [11–13].

Phage infection initiates through an interaction of the phage with bacterial cell surface-exposed molecular receptors, such as lipopolysaccharide (LPS) moieties, peptidoglycan, teichoic acids, capsules, or proteinaceous components. In the case of T4, infection starts with the molecular recognition and reversible binding between adhesin proteins at the tip of the long tail fibres of the phage and the receptors in the outer layer of the *E*. *coli* cell surface [14]. T4 uses both the outer membrane protein C (OmpC) and LPS as coreceptors: if OmpC is present, as in *E*. *coli* K-12, T4 targets OmpC as a first receptor to recognise the host [14] and infects regardless of the terminal sugar residue of LPS. In the absence of OmpC, as in *E*. *coli* B, T4 attaches to LPS chains with an exposed terminal glucose residue [15]. These initial reversible bindings trigger a series of conformational changes of the tail baseplate that permits T4 short tail fibres to irreversibly bind to the lipid A keto-deoxyoctulosonate region of LPS. Such baseplate conformational changes also trigger a contraction of the tail sheath [16], followed by the penetration of the tail tube into the bacterial cytoplasm and T4 DNA ejection into the cell cytoplasm [17]. This is followed by a rapid shut off of DNA replication, transcription and protein synthesis in *E*. *coli* [18]. Meanwhile, the phage genome replication is initiated, followed by gene expression, protein assembly, packaging of completed phage particles, and ultimately bursting of the host *E*. *coli* cell approximately 25 min postinfection [9].

This molecular understanding was obtained via experiments carried out in well-mixed cultures, where *E*. *coli* rapidly evolved resistance to phages [19], albeit at a fitness cost that varied dependent on the type of mutation [20,21], the environmental temperature, and the availability of nutrients and water [22–25]. While these population-level studies in spatially unstructured environments suggested that evolved cultures are relatively homogeneous [19], the population and evolutionary dynamics of phage–bacteria interactions are likely more complex in natural environments. For example, when *Pseudomonas fluorescens* growing in a soil community was exposed to the lytic bacteriophage SBW25ϕ2, bacteria were more resistant to their contemporary than to past or future phages. This was in contrast to coevolution in vitro, which was characterised by an increase in resistance over time [26]. Moreover, de novo evolution of resistance to the lytic phage FRS in *Pseudomonas syringae* was negligible in planta despite high levels of resistance evolution in vitro [27]. This may be because most natural environments, such as biofilms, soil, or the tissues of plants and animals, possess a spatial structure [28].

Studies that examined phage–bacteria interactions in spatially structured (semi)natural or artificial environments often report that genetic resistance to phages evolves at much lower frequencies than those observed in mixed broth [29–35]. One explanation is that bacteria form biofilms on solid surfaces that shield cells on the inside from phages [34,35]. If so, disrupting biofilms may be an important factor in the success of phage therapy in clinical settings. However, it is also possible that the persistence of sensitive bacteria is due to less efficient phage amplification in structured environments, the existence of permanent or ephemeral spatial refuges for bacteria to hide in, and resistant genotypes not invading as efficiently as they would in a mixed environment [36]. Here, we fill this fundamental gap in our knowledge of phage–bacteria interaction by using single-cell microfluidics that has recently unlocked a new era of virology [37,38] but has thus far been rarely applied for studying phage–bacteria interaction [39].

**Fig 1:**
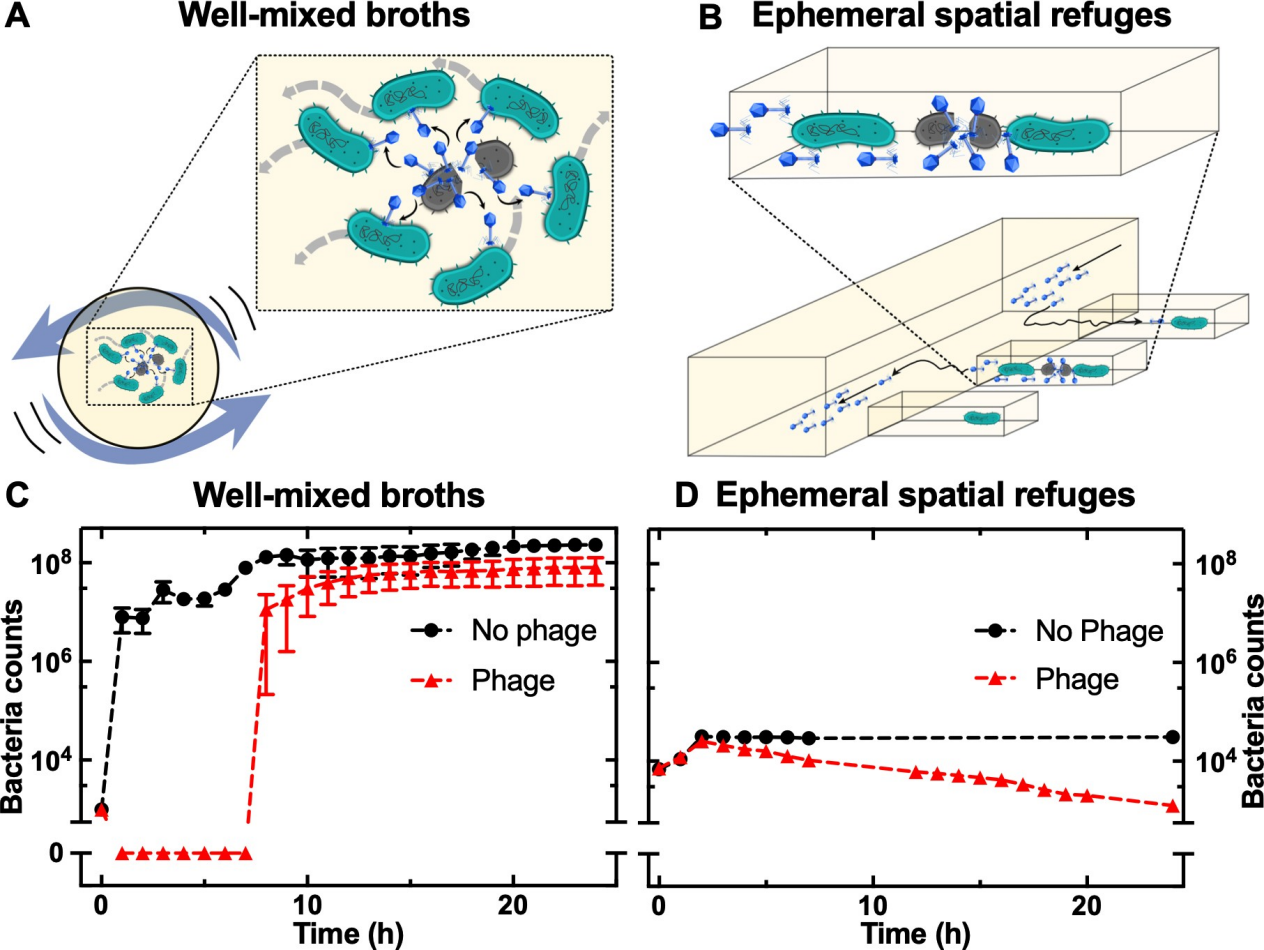
The structure of the environment affects the bacterial population dynamics in response to phage. Schematics illustrating T4 epidemics in *E*. *coli* in (A) well-mixed microwells and (B) microfluidic devices structured with ephemeral spatial refuges. Corresponding temporal dependence of bacterial population size in (C) the unstructured and (D) the structured environment in the presence (triangles) and absence (circles) of phage T4. Data are the mean and standard error of the mean of at least 4 biological replicates in the unstructured environment and of 200 spatial refuges in biological triplicate in the structured environment. Error bars are hidden behind the corresponding data points in the structured environment measurements due to the large statistical samples. It is noteworthy that similar initial bacterial population sizes were used in the unstructured and structured environments; compare data at t = 0. Dashed lines are guides for the eye. Numerical values for each replica in Fig 1C and 1D are provided in Data B and Data F in S1 File, respectively.

We introduce a high-throughput, microfluidics-based platform to perform kinetic analysis of phage infections in individual bacteria cultured as planktonic cells with or without spatial refuges (Fig 1A and 1B, respectively), in the absence of biofilm formation.

In the absence of spatial refuges, exposure of *E*. *coli* to T4 resulted in very rapid culture collapse followed by regrowth due to the emergence of genetic resistance to T4. In stark contrast, in ephemeral refuges, the bacterial population initially expanded, followed by a reduction in the population with some bacteria surviving phage without the emergence of genetic resistance mechanisms. These bacterial population dynamics reveal the intrinsic heterogeneity in response to phage infection, due to the appearance of phenotypic variants, either in a replicative or nongrowing state. Our experimental data and agent-based simulations show that phenotypic variants with low phage receptor expression are selected in structured environments because phages preferentially infect bacteria displaying abundant phage receptors.

Our study represents a stepping stone for understanding how the ecological context affects bacteria–phage interactions down to the individual cell level and how this drives the evolutionary dynamics of bacteria and phage populations. This information will be crucial for establishing lytic bacteriophages as a viable therapy, for the development of new biotechnological tools, and to predict how phage could shape the ecology and evolution of microbial communities and ecosystem-level processes.

## Results

### The environmental structure affects bacterial population dynamics during T4 infection

In well-mixed environments (i.e., shaken 200 μl wells of a 96-microwell plate; Fig 1A) T4 rapidly amplified, as expected, in the presence of *E*. *coli* BW25113 (S1 Fig and Data A in S1 File). This resulted in a rapid collapse of the bacterial population, followed by regrowth of the culture to a final density comparable to bacteria growing in the absence of phages (triangles and circles, respectively, in Fig 1C and Data B in S1 File), suggesting the emergence of genetically resistant mutants that could either be preexistent in the initial inoculum or have evolved during exposure to phage. This finding was obtained using 10^3^ bacteria as the initial inoculum in each microplate well and a multiplicity of infection (MOI) of 1. To test for the effect of population size on the emergence of resistance, we repeated this experiment with different initial population sizes while maintaining the MOI constant at 1. Similar bacterial population dynamics were measured when the initial bacterial population was above 10^3^ bacteria per well, whereas the bacterial population became extinct when the initial inoculum was below 10^3^ bacteria per well (S2 Fig and Data C in S1 File) in accordance with previous findings [34].

These findings suggest that genetically resistant mutants were preexistent in the initial inoculum and emerged during exposure to phage. We further verified that these bacterial population dynamics were not dictated by the small liquid volumes and reduced bacterial and phage numbers by measuring similar population dynamics in shaken flasks containing 100 ml liquid cultures (S3 Fig and Data D in S1 File). Furthermore, by varying the bacterial concentration, while keeping the phage concentration constant at 10^*7*^ ml^*−1*^, we measured similar bacterial population dynamics in response to different MOIs (S4 Fig and Data E in S1 File). By sequencing the bacterial culture following 24 h of exposure to phages, we found a single nucleotide polymorphism (SNP) in the *fliR*/*rcsA* intergenic region (S1 Table). The *rcs* signal transduction pathway contributes to envelope stress response by monitoring the cell surface composition [40]. This pathway regulates several genes including those involved in the synthesis of colanic acid capsular polysaccharide [40]. RcsA has recently been identified as one of the top candidates that increased *E*. *coli* fitness in the presence of several phages, including T4 [41–44], by inducing the capsule synthesis gene cluster that triggers the overproduction of colanic acid [40]. Furthermore, we identified additional mutations likely hitchhiking with the capsule associated SNP, as they were either present after 6 h but not after 24 h exposure to T4, or were present at low frequencies (S1 Table).

In order to contrast these population dynamics with the interactions between *E*. *coli* and T4 in homogeneous but ephemeral spatial refuges, we introduced a high-throughput, microfluidics-based platform to perform kinetic analysis of phage infections in individual bacteria. We used a microfluidic mother machine device [45] equipped with thousands of compartments (i.e., channels of dimensions comparable to those of individual *E*. *coli* cells), each initially hosting on average one bacterium with an overall initial bacterial population of 10^*3*^ as in the microwell plate experiments above (compare initial populations at t = 0 in Fig 1C and 1D). However, the key difference compared to a microwell is that the compartments in the mother machine are physically separated from each other [46], thus preventing both the mixing of the bacterial population and the direct propagation of phage from an infected and lysed bacterium to all its surrounding neighbours (Fig 1B), a situation that is encountered in microwells and flasks (Fig 1A). Furthermore, the surfaces of such compartments were coated with bovine serum albumin to prevent cell attachment, thus maintaining bacteria in a confined but planktonic state. Indeed, bacteria wriggled around their position during our time-lapse microscopy imaging but did not leave their hosting channels due to physical confinement and the reduced motility of the *E*. *coli* BW25113 strain. In control experiments loaded with *E*. *coli* in the absence of phage, the population size expanded 5-fold during the first 2 h of incubation in Lysogeny broth (LB). Afterwards, the population reached a plateau for the next 22 h of incubation in LB (circles in Fig 1D and Data F in S1 File) since each channel could only physically accommodate up to 6 *E*. *coli* at one time and the newest progeny at each channel entrance was flushed in the mother machine outflow. These data confirmed robust growth of *E*. *coli* in the mother machine device as previously reported [45,47]. Indeed, nutrients, oxygen, and metabolic waste diffuse in and out the ephemeral refuges [45] where hosted bacteria do not experience any additional stress compared to growth in microwells and flasks.

Surprisingly, we measured a comparable initial expansion of the *E*. *coli* population per channel in separate experiments when phages and bacteria where simultaneously added to the mother machine at t = 0 as in the microwells and flasks experiments above. A constant phage supply was then maintained throughout the experiments in the mother machine and averaging measurements from biological triplicate we found that the bacterial population grew from 7,110 ± 40 bacteria at t = 0 to a maximum of 25,540 ± 310 bacteria following 2 h of phage exposure (triangles in Fig 1D) despite the presence of phages. Moreover, over 70% of the bacteria were able to divide at least once before lysis within the 24-h exposure to phage (S5A Fig and Data G in S1 File). These population dynamics in the presence of ephemeral spatial refuges were in striking contrast with the rapid collapse of the bacterial population upon exposure to T4 in microwells or flasks (Figs 1C, S3 and S4).

These different bacterial population dynamics could not be ascribed to phages not reaching the spatial refuges. In fact, fluorescent nanospheres (of dimensions similar to bacteriophage T4) reached 99% of the spatial refuges within 1 h post-addition to the mother machine (with a mean of 1.2 ± 0.7 particles per channel h^−1^; S6A Fig and Data H in S1 File). Moreover, the median residence time was 40 s for each particle in each refuge (S6B Fig and Data I in S1 File), each particle exploring on average the length of each refuge. Therefore, it is reasonable to assume that during the 24-h long phage infection assay, each spatial refuge was explored, on average, by 24 phages, although we cannot exclude that a minority of survivors do not encounter phages due to the presence of spatial refuges.

### The phage concentration affects the bacterial population dynamics in the presence of spatial refuges

**Fig 2:**
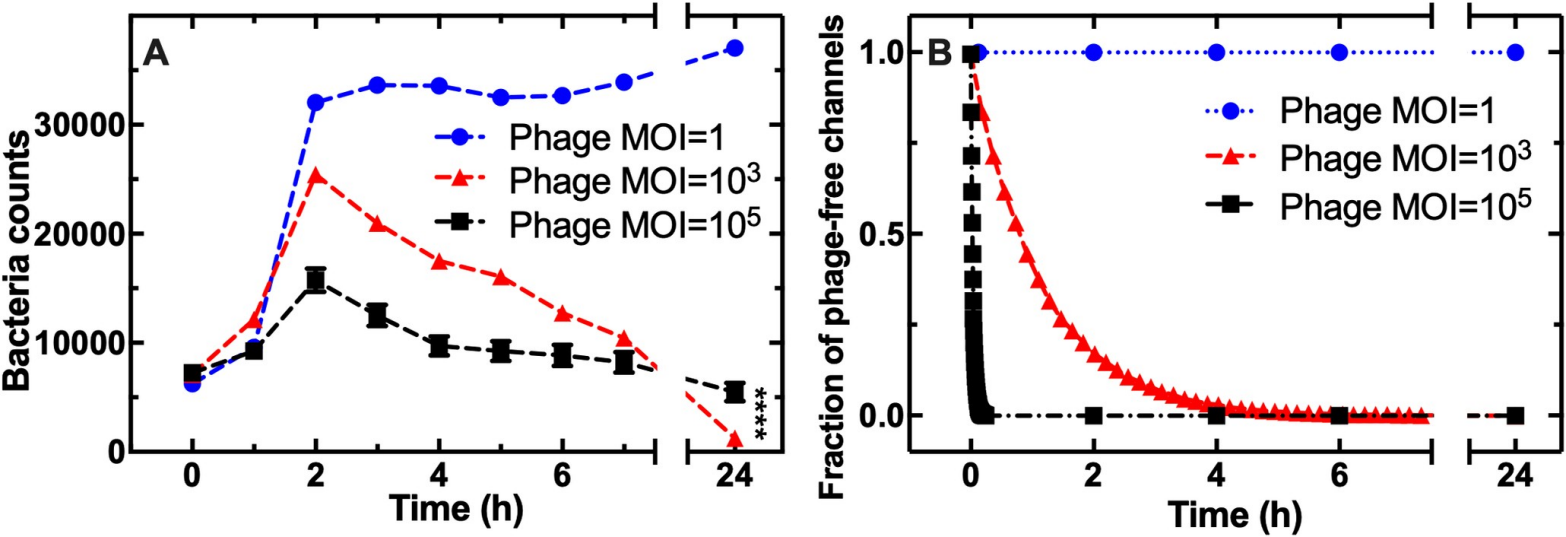
The phage concentration affects the bacterial population dynamics in the structured environment. **(A)** Temporal dependence of the bacterial population size with T4 phages continuously injected in the mother machine device from t = 0 onwards at an MOI of 1 (circles), 10^*3*^ (triangles), or 10^*5*^ (squares). This MOI refers to the concentrations of phage and bacteria in the entire device rather than in each individual compartment. Data and error bars are the average and standard error of the mean of 200 single-compartment measurements in biological triplicate. The dashed lines are guides for the eye. Some of the error bars are hidden behind the corresponding data points. **** indicates a *p*-value ≤ 0.0001. **(**B**)** Corresponding simulated temporal dependence of the fraction of phage-free refuges up to time *t*. Data points were obtained via Lattice–Boltzmann simulations, and statistics was collected from over 2,600 simulated phages in a periodic cross-section of the mother machine. The dashed lines are guides for the eye. Numerical values for each replica in Fig 2A and 2B are provided in Data J and Data K in S1 File, respectively. MOI, multiplicity of infection.

By altering the concentration of phages added into the mother machine, we manipulated the bacterial population dynamics. We observed complete survival at a low phage density (nominal MOI of 1, i.e., comparing the initial bacterial and phage population size rather than the number of bacteria and phages in each refuge; blue circles in Fig 2A and Data J in S1 File).

On the other hand, during the first 2 h of treatment at a nominal MOI of 10^*5*^, the bacterial population expanded only 2-fold, whereas it expanded 4-fold for treatment at a nominal MOI of 10^*3*^ (t = 2 h squares and triangles, respectively, in Fig 2A). This was due to 50% of the bacteria lysing without dividing at a nominal MOI of 10^*5*^ (S5B Fig and Data G in S1 File). These experimental data were in accordance with Lattice–Boltzmann simulations, which predicted that the fraction of compartments that were not reached by at least 1 phage in the first 2 h of exposure to T4 was close to 1 for a nominal MOI of 1, and close to 0 for a nominal MOI of 10^*3*^ and 10^*5*^ (Fig 2B and Data K in S1 File). These Lattice–Boltzmann simulations also predicted that under an applied flow rate of 100 μl h^−1^ on average 1.5 ± 0.1 particles h^−1^ reached each compartment in accordance with the nanosphere measurements above and that in the absence of flow a slightly larger amount of particles (2.0 ± 0.2 particles h^−1^) reached each compartment (S7 Fig and Data L in S1 File).

After the first 2 h of exposure to phage, we observed a steady decline in the bacterial population in the structured microfluidic environment at both a nominal MOI of 10^*3*^ and 10^*5*^ (Fig 2A). Taken together, these findings suggest that the different bacterial population dynamics between unstructured and structured environments were not due to reduced phage infectivity in the latter environment, but rather to the presence of ephemeral spatial refuges where bacteria could transiently hide from phages in the structured environment. It is also worth noting that we did not find any significant difference in bacterial death along the length of the microfluidic compartments, in accordance with previous studies showing that the diffusion of molecules (i.e., phage particles in this study) is isotropic across the length of these compartments [45,47]. Finally, we demonstrated a significantly lower killing by phages for the treatment with an MOI of 10^*5*^ compared to an MOI of 10^*3*^ (5,670 ± 630 and 1,280 ± 180 bacteria, respectively, t = 24 h squares and triangles in Fig 2B, *p*-value < 0.0001), suggesting that higher phage doses do not necessarily imply greater bacterial clearance.

### The environmental structure affects bacteria–phage encounter rate dynamics

**Fig 3:**
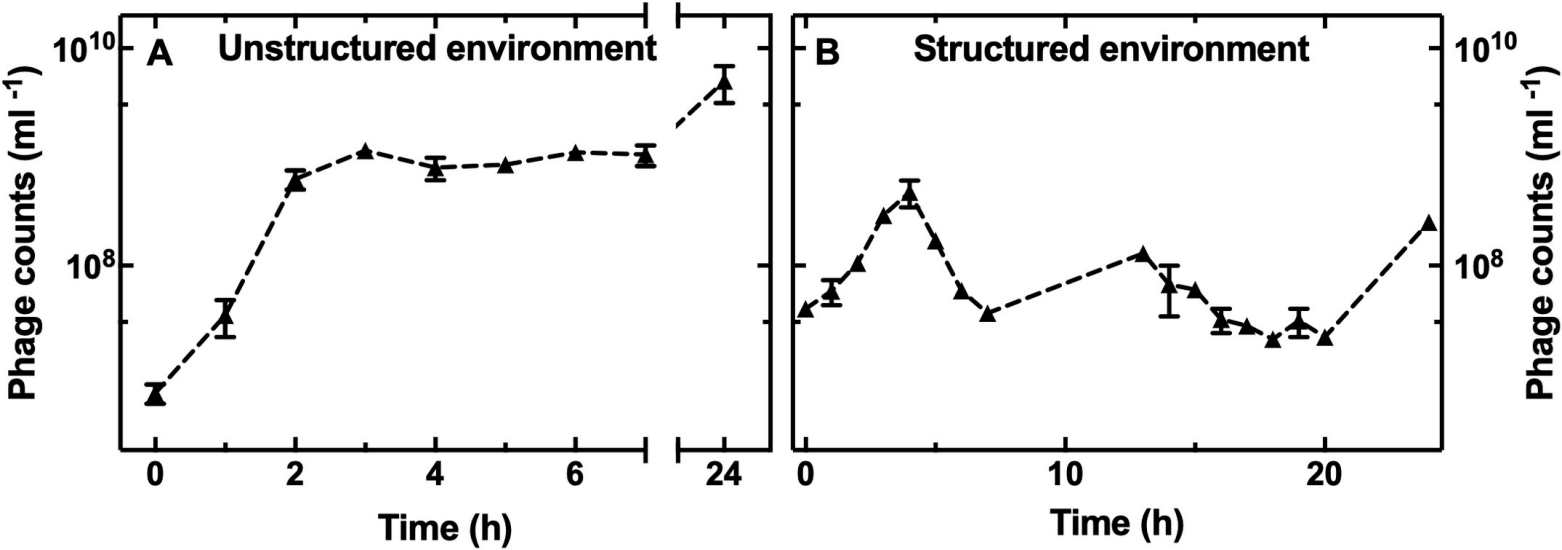
The structure of the environment impacts the phage population dynamics. Temporal dependence of the concentration of phages infecting *E*. *coli* in **(A)** the unstructured and **(B)** the structured environment. Data are the mean and standard error of the mean of biological triplicates. Dashed lines are guides for the eye. Some of the error bars are hidden behind the corresponding data points. The corresponding bacteria population dynamics are reported in S4 Fig (MOI = 1, blue circles) and Fig 1D (red triangles), respectively. Numerical values for each replica are provided in Data M in S1 File. MOI, multiplicity of infection.

To gain a better understanding of the phage population dynamics, the outflow from the mother machine outlet was collected, and the phage density was measured over time and compared to that found in infection experiments in the unstructured environment. After 2-h addition of T4 to *E*. *coli* growing in the unstructured environment, the phage concentration increased 100-fold (Fig 3A and Data M in S1 File), whereas the outflow concentration of phage increased by only a factor of 3 in the structured microfluidic environment (Fig 3B and Data M in S1 File).

This shows that phage amplification is much less efficient in the structured environment due to the presence of ephemeral spatial refuges, even if the phages can self-propagate under those conditions. This further demonstrates that the phages maintain their infectivity within the microfluidic device. In fact, the phage concentration in the structured environment steadily increased up to 5 × 10^*8*^ ml^*−1*^ at t = 4 h post-addition to the spatial refuges and then fluctuated between 5 × 10^*7*^ and 10^*8*^ ml^*−1*^ for the subsequent 20 h (Fig 3B), while the bacterial population steadily decreased (Fig 1D).

Using these data, we could estimate bacteria–phage encounter rates. In the unstructured environment, the encounter rate can be estimated as follows [48]:

$$E=C_{B}C_{P}k,$$

where *C*_*B*_ and *C*_*P*_ are the bacterial and phage concentrations, respectively, whereas *k* is the diffusion limited rate for small particles (phage) captured upon contact with a spheroidal absorber (bacterial cell) and can be calculated as follows [48]:

k=2πDl/ln(2l/w),

where *D* is the phage diffusivity, and *l* and *w* are the bacterial length and width, respectively. Taking *D* = 4 μm^2^ s^−1^ and the typical dimensions of *E*. *coli* in our study (*l* = 3 μm and *w* = 0.8 μm), we obtained *k* = 2.2 × 10^−9^ ml min^−1^, which is in agreement with previously reported T4 infection rates for *E*. *coli* [49]. Considering that at t = 0 the phage and bacterial concentrations in the unstructured environment are approximatively 10^*7*^ ml^*−1*^ (Fig 3A and blue circles, MOI = 1, in S4 Fig), the estimated encounter rate was 1.2 × 10^*7*^ encounters ml^*−1*^ h^−1^. Therefore, on average, each bacterium encountered at least 1 phage within the first hour of bacteria and phage addition to the unstructured environment. Similarly, in the structured environment, each bacterium encountered at least 1 phage within the first hour of phage and bacteria injection in the mother machine according to our experiments carried out using nanospheres of dimensions similar to T4 (mean of 1.2 ± 0.7 particles per channel per hour; S6A Fig). However, bacteria–phage encounter rates became dramatically different between the 2o different environments from t = 2 h onwards. In the unstructured environment, the phage concentration increased to around 10^*9*^ ml^*−1*^ at t = 2 h, whereas the bacterial concentration decreased to around 10^*4*^ ml^*−1*^ (Fig 3A and blue circles, MOI = 1, in S4 Fig). As a result, the predicted encounter rate was 2.4 × 10^*6*^ encounters ml^*−1*^ h^−1^, each bacterium encountering more than 200 phages per hour. In the structured environment, in contrast, both the phage and bacteria concentrations increased of a factor of 3 at t = 2 h (Fig 3B and red triangles in Fig 1D); therefore, the bacteria–phage encounter rate remained constant at around 1 encounter per hour.

We also verified that the measured phage counts in the outflow of control mother machine experiments in the absence of bacteria or in the presence of bacteria that could not be infected by phage (i.e., bacteriophage T3) was equal to the phage input (S8A and S8B Fig, respectively, Data N in S1 File) and rapidly decreased when phages were no longer added to the device input (t = 2 h in S8A Fig). These data demonstrate both phage viability in the mother machine and that any phage binding to the microfluidic device did not affect the number of phage available for infection. Finally, we did not observe phage amplification in the presence of spatial refuges for the treatment with an MOI of 10^*5*^ (S9 Fig and Data O in S1 File), this explaining the abovementioned lower killing by phages for the treatment with an MOI of 10^*5*^ compared to an MOI of 10^*3*^.

### Phenotypic resistance to T4 in the presence of spatial refuges

Despite phage amplification by t = 24 h, *E*. *coli* were capable of surviving exposure to T4 both in the absence or presence of ephemeral refuges, but via 2 orthogonal strategies. In the absence of ephemeral refuges, changes to the cell surface (S1 Table) conferred genetic resistance to T4 with the bacterial population expanding up to a maximum of 8 × 10^*7*^ cells (t = 24 h, triangles in Fig 1C). In the presence of ephemeral refuges, the bacterial population was not completely eradicated but reduced to a minimum of 1,280 – 180 bacteria (t = 24 h, triangles in Fig 1D), revealing population subsets that survived because were phenotypically resistant to T4, although we cannot exclude that a minority of these survivors did not encounter T4 due to the presence of spatial refuges. However, these surviving subsets were not genotypically resistant to T4 since none of the bacteria collected in the outflow of the mother machine grew on LB agar plates containing T4 (S10 Fig) and no SNPs were identified in these samples.

Taken together, these data suggest that the ecological context affects the outcome of phage treatment. In fact, selection for genetic resistance to phage in the presence of ephemeral spatial refuges is relaxed compared to well-mixed environments, due to reduced bacteria–phage encounter rates in the presence of spatial refuges. Importantly, the lack of emergence of genetic resistance to T4 in the structured environment could not be ascribed to the number of bacteria in the microfluidic device since a similar sized bacterial population was capable of evolving genetic resistance in shaken microwells (t = 0 in Fig 1C and 1D).

**Fig 4:**
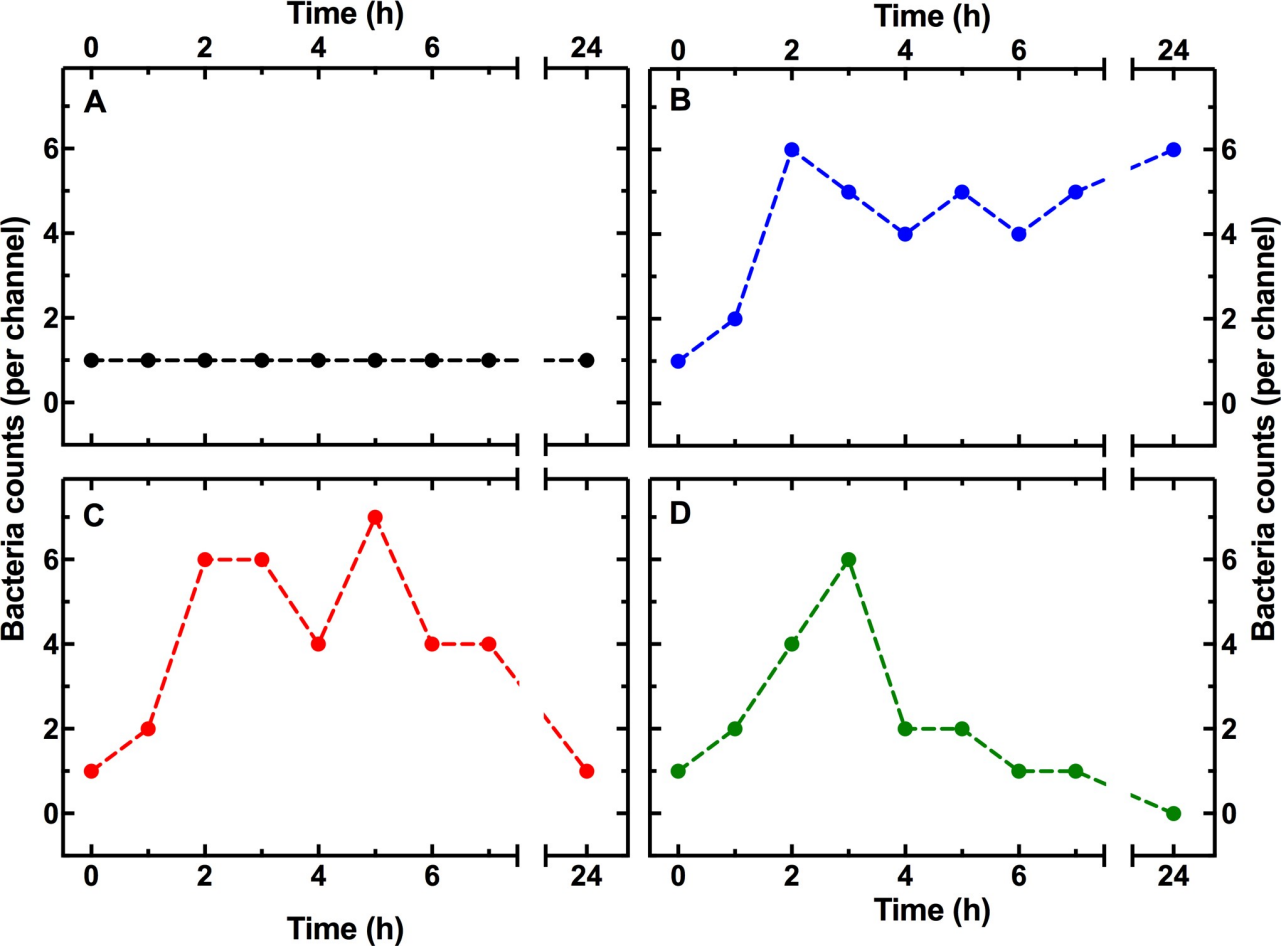
Dissecting *E*. *coli* phenotypic resistance to T4. Representative temporal dependence of bacterial density per compartment for a bacterium that **(A)** remains viable but nongrowing as determined by live/dead staining at t = 24 h, **(B)** duplicates and remains alive together with all its progeny, **(C)** duplicates but some of its progeny lyses, and **(D)** filaments upon exposure to T4 phage. Measurements were carried out in 200 channels of the structured mother machine environment in biological triplicate. Dashed lines are guides for the eye. Numerical values are provided in Data P in S1 File.

We then investigated the mechanisms underlying the phenotypic resistance of *E*. *coli* to T4 in the presence of ephemeral spatial refuges. In order to do this, we followed the fate of individual bacteria throughout the whole duration of our mother machine experiments (representative fate trajectories in Fig 4 and Data K, Data P, and Data Q in S1 File). We found that some bacteria rapidly lysed following exposure to phage, leaving cellular debris, and empty microfluidic channels (S11A Fig), others lysed after several hours of phage supply to the microfluidic device without any cell division (S11B Fig), with one or with multiple cell divisions (S11C and S11D Fig, respectively).

Crucially, we identified 3 main phenotypic survival strategies to T4. First, we observed that some bacteria were not killed during phage treatment and did not grow (Fig 4A).

Second, we observed bacteria that grew and divided during exposure to T4. Some of these bacteria survived with all their progeny (Fig 4B). For others, one or more of their progeny was killed by T4 (Fig 4C). Third, we observed bacterial filamentation in response to T4 exposure (Fig 4D) but not in control experiments in the absence of T4. The relative number of filamenting cells increased with time up to a maximum of 0.26 ± 0.01 at t = 7 h (S12 Fig and Data R in S1 File).

The majority of these survivors must have encountered phages considering that (i) nanospheres of dimensions similar to bacteriophage T4 reached 99% of the spatial refuges within 1 h post-addition to the mother machine (S6 Fig) with a predicted encounter rate of 1 per hour, and this was further confirmed via Lattice–Boltzmann simulations (Fig 2B); and (ii) some of their surrounding clones lysed (S13 Fig), releasing around 100 new viral particles per cell (S1 Fig) in their immediate proximity. However, these cells were not genetically resistant since the mother machine outflow was readily killed by T4 as described above (S10 Fig).

### Heterogeneity in the expression of the phage receptor contributes to phenotypic resistance in the presence of spatial refuges

**Fig 5:**
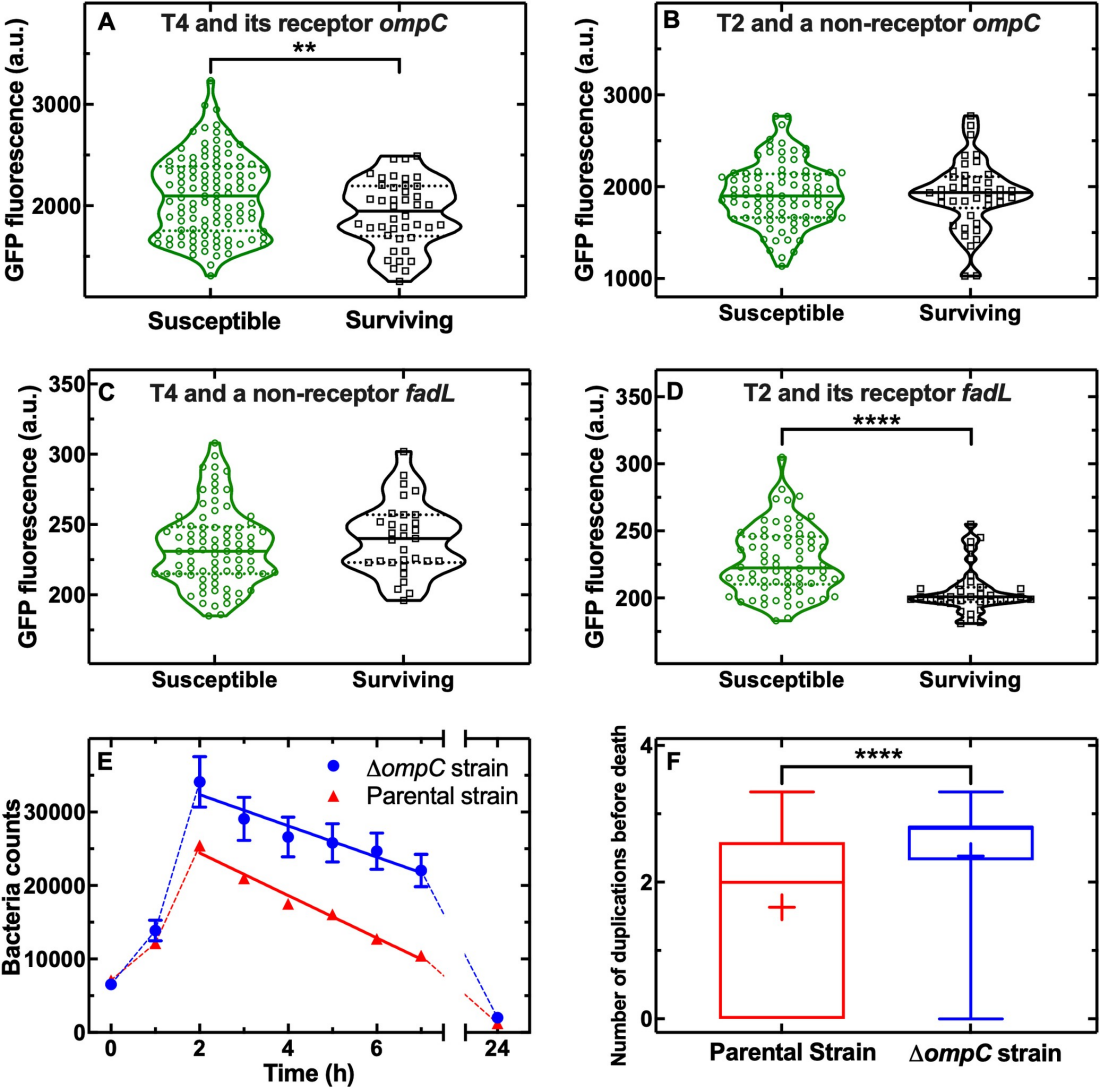
Phenotypic heterogeneity in phage receptor contributes to phenotypic resistance to phage in the presence of ephemeral spatial refuges. Distributions of GFP levels reporting on the expression of the T4 receptor OmpC before exposure to **(A)** T4 or **(B)** T2 phage in *E*. *coli* that later lysed (green violin plot) or survived (black violin plot) exposure to phage. Data were obtained in biological triplicate for a total of *N* = 108, *N* = 42 bacteria that were killed or survived T4 exposure, respectively, and *N* = 85, *N* = 42 bacteria that were killed or survived T2 exposure, respectively. Distributions of GFP levels reporting on the expression of the T2 receptor FadL before exposure to **(C)** T4 or **(D)** T2 phage in *E*. *coli* that later lysed (green violin plot) or survived (black violin plot) exposure to phage. Data were obtained in biological triplicate for a total of *N* = 82, *N* = 31 bacteria that were killed or survived T4 exposure, respectively, and *N* = 72, *N* = 35 bacteria that were killed or survived T2 exposure, respectively. **(E)** Temporal dependence of mean bacterial population size for the parental strain (triangles) and a Δ*ompC* deletion mutant (circles) upon exposure to T4 phage. Data are the mean and standard error of the mean of 200 single-compartment measurements in biological triplicate. Some of the error bars are hidden behind the corresponding data points. Solid lines are linear regression fitting to the data returning an x-intercept of 10 and 17 h for the parental and Δ*ompC* strain, respectively. Dashed lines are guides for the eye. **(f)** Distributions of number of generations before death caused by T4 for the parental (red) and Δ*ompC* strain (blue boxplot). The bottom and top of the box are the first and third quartiles, the line and plus symbol inside the box are the median and mean, the bottom and top lines outside the box represent the 10th and 90th percentiles, respectively. Data were obtained in biological triplicate. ** indicates a *p*-value ≤ 0.01, **** indicates a *p*-value ≤ 0.0001. Numerical values for Fig 5A–5D are provided in Data S in S1 File. Numerical values for Fig 5E and 5F are provided in Data V and Data W in S1 File. GFP, green fluorescent protein; OmpC, outer membrane protein C.

We then hypothesised that the observed heterogeneity in *E*. *coli* response to T4 was due to cell-to-cell differences in the expression of one of the key surface receptors for T4, namely OmpC [50]. When we used a green fluorescent protein (GFP) reporter strain for *ompC* expression in the presence of ephemeral spatial refuges, we found that the subpopulation of *E*. *coli* that was killed by T4 by 24 h (*N =* 108 susceptible cells) displayed an initial distribution of GFP fluorescence (at t = 0) that was significantly higher than the one measured for the subpopulation of *E*. *coli* that survived T4 treatment (*N* = 42 surviving cells, Fig 5A, *p*-value = 0.006, **, Data S in S1 File).

In contrast, when the *E*. *coli* reporter strain for *ompC* was exposed to phage T2, which uses FadL and OmpF, rather than OmpC, as receptors [44], the subpopulation of *E*. *coli* that was killed by T2 by 24 h displayed an initial distribution of GFP fluorescence that was similar to that measured for the subpopulation of *E*. *coli* that survived T2 treatment (*N =* 85 and 42 susceptible and surviving cells, respectively, Fig 5B, *p*-value = 0.93, not significant). Furthermore, when the *E*. *coli* reporter strain for *fadL* was exposed to phage T4, the subpopulation of *E*. *coli* that was killed by T4 by 24 h displayed an initial distribution of GFP fluorescence that was similar to that measured for the subpopulation of *E*. *coli* that survived T4 treatment (*N =* 82 and 31 susceptible and surviving cells, respectively, Fig 5C, *p*-value = 0.28, not significant). In contrast, when the *E*. *coli* reporter strain for *fadL* was exposed to phage T2, the subpopulation of *E*. *coli* that was killed by T2 by 24 h displayed an initial distribution of GFP fluorescence that was significantly higher than the one measured for the subpopulation of *E*. *coli* that survived T2 treatment (*N* = 72 and 35 susceptible and surviving cells, respectively, Fig 5D, *p*-value < 0.0001, ****).

To further verify that the observed heterogeneity in GFP fluorescence was not due to cell-to-cell differences in plasmid copy numbers or background fluorescence [51], we carried out control experiments using T4 and an *E*. *coli* strain harbouring the same GFP-encoding plasmid as the *ompC* and *fadL* reporter strains but without encoding any promoter (i.e., the promoterless plasmid pUA66 from the transcriptional reporter library described in [52], expected to display homogeneously low levels of background fluorescence due to transcriptional read-through [53]). Accordingly, we found that the subpopulation of *E*. *coli* that was killed by T4 by 24 h displayed an initial distribution of GFP fluorescence (at t = 0) that was similar to the one measured for the subpopulation of *E*. *coli* that survived T4 treatment (*N* = 54 and 17 susceptible and surviving cells, respectively, S14 Fig, *p*-value = 0.62, not significant, Data T in S1 File). These data confirm that the cell-to-cell differences in GFP fluorescence measured when using the *ompC* and *fadL* reporter strains are not due to plasmid copy numbers or background fluorescence. We further verified that the different *E*. *coli* strains employed displayed comparable growth at the population level in the unstructured environment (S15 Fig, Data U in S1 File).

Furthermore, when we performed the single-cell infection assay in the presence of ephemeral spatial refuges with an *E*. *coli ompC* deletion mutant, we found an increase in the bacterial population compared to the parental strain with a maximum of 34,750 ± 1,430 at t = 2 h (Fig 5E and Data V in S1 File). This population increase was likely due to a significant increase in the mean number of divisions, with fewer cells lysing after 0 or 1 divisions (mean division number before death of 1.6 and 2.4 for the parental and Δ*ompC* strain, respectively, Fig 5F and Data W in S1 File). This may be due to phages binding to the bacterial surface less efficiently since the T4 receptor, OmpC, had been removed. This reduction in binding sites for T4 may also have led to a delay in the predicted extinction time of the mutant compared to the parental strain (17 and 10 h after T4 addition, respectively; Fig 5E). Collectively, these data suggest that in the presence of spatial refuges, phages select for bacteria with low expression of phage surface receptor by preferentially infecting and killing bacteria displaying high expression of phage receptors.

In order to explore these findings further, we developed a mathematical modelling framework to track the population dynamics of bacteria resistance to phage in 2 different environments: structured and unstructured. In this model, bacteria are characterised by 2 independent traits, genetic resistance and phenotypic resistance associated with surface receptor expression levels. Structure is introduced by subdividing the population into a large number of separate channels, each with a relatively small carrying capacity, similar to our experimental setup (see Methods for full model details). We simulated this model in the absence of structure (akin to well-mixed broth, with a carrying capacity of 10^*6*^ bacteria) and in the presence of structure (akin to ephemeral spatial refuges, with an overall carrying capacity of 4 × 10^*4*^ bacteria) using a secondary infection rate of *R* = 20 (i.e., the average number of secondary infected bacteria that can arise per infected cell assuming all cells are susceptible). Note, the qualitative dynamics presented here are independent of the value of *R*, which mostly affects the speed at which selection for resistance occurs.

**Fig 6:**
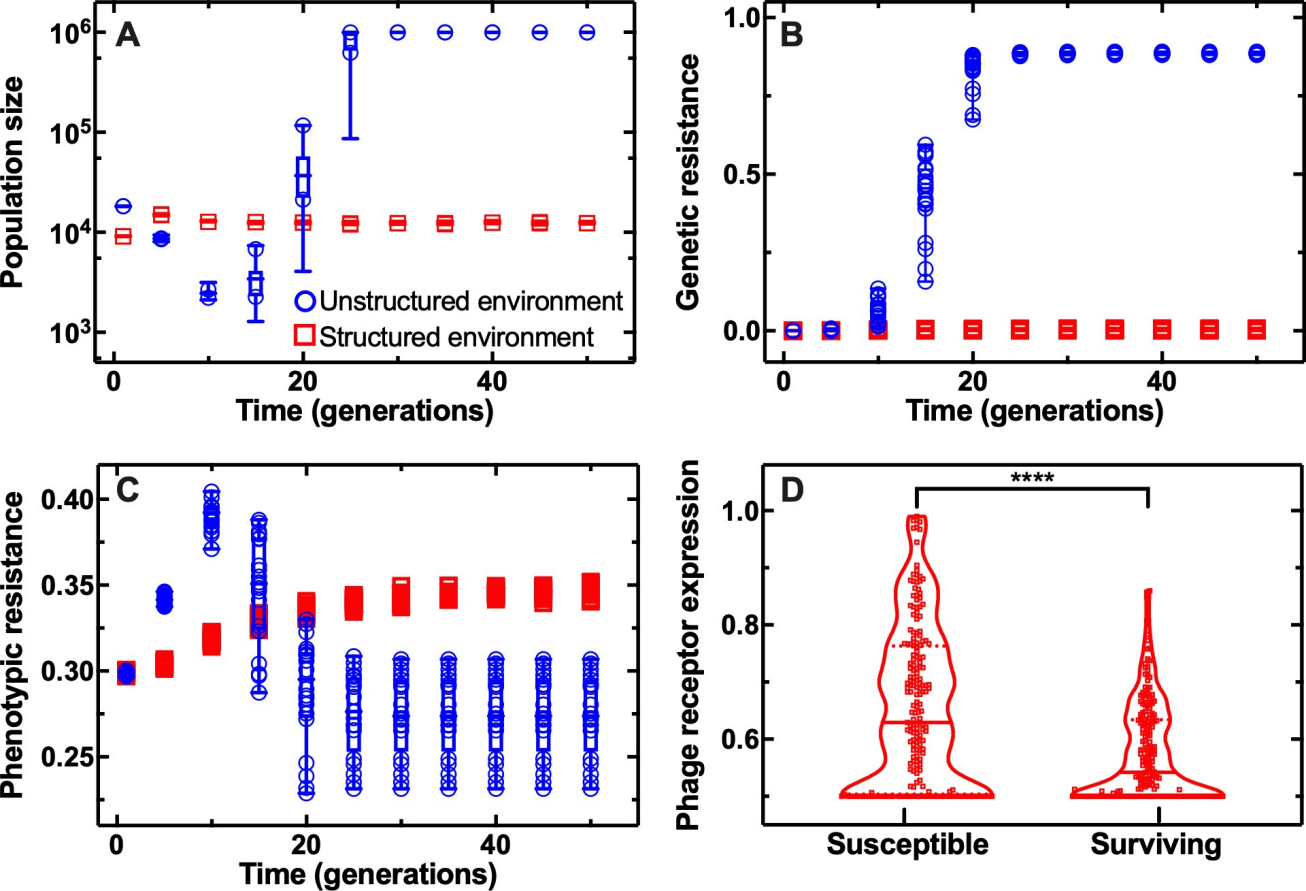
Effect of the structure of the environment on bacterial population dynamics during phage infection according to agent-based simulations. Temporal dependence of **(A)** the bacterial population size, the fraction of the bacterial population **(B)** genetically or **(C)** phenotypically resistant to phage in the unstructured and structured environment (blue circles and red squares, respectively). Data were obtained for 20 independent model simulations. **(D)** Distributions of phage receptor levels in bacteria that were susceptible to or surviving phage at 25 generations post-phage addition to the structured environment. Data were obtained for 200 randomly sampled cells. **** indicates a *p*-value ≤ 0.0001. Numerical values for each replica in Fig 6A–6C are provided in Data X in S1 File. Numerical values for Fig 6D are provided in Data Y in S1 File.

As shown in Fig 6, the model simulations are well aligned with our experimental findings, showing that in the presence of phage, the bacterial population expands to a greater extent in the unstructured environment compared to the structured environment (Fig 6A and Data X in S1 File) due to the emergence and rapid selection of genetic resistance only in the unstructured environment (Fig 6B).

Moreover, these simulations revealed that in both environments, the average phenotypic resistance to phage, linked to phage receptor expression, increased over time (Fig 6C) due to selection of phenotypic variants that survive phage in the absence of genetic mutations. In the structured environment, the average phenotypic resistance to phage plateaued from 20 generations onwards. In the unstructured environment, the average phenotypic resistance started to decrease after 20 generations (Fig 6C) due to the emergence of genetic resistant mutants (Fig 6B) with full protection against phage infection independently of surface receptor expression.

Finally, our agent-based simulations revealed that after 25 generations, phages preferentially infected bacteria displaying abundant surface receptors (Fig 6D and Data Y in S1 File) in accordance with our experimental data (Fig 5A and 5D).

## Discussion

The ecology and evolution of microbial communities are shaped by bacteria–phage interactions, and there is growing evidence that coevolution favours phenotypic and genetic diversities [1,54,55]. A diverse arsenal of genetic mechanisms permitting bacteria to resist temperate and lytic phages have been reported [7,8,56]. In contrast, phenotypic mechanisms have been reported only for bacterial survival to temperate phages [39,57,58], whereas phenotypic responses to virulent phages remain to be investigated. This fundamental gap, in our knowledge, is partly due to the fact that bacteria–phage interactions have traditionally been investigated via population-level studies that do not permit the dissection of phenotypic heterogeneities within populations. Here, we fill this fundamental gap by demonstrating that in contrast with known genetic resistance mechanisms in well-mixed environments [41,42,59], *E*. *coli* display 3 previously unrecognised short-term phenotypic resistance strategies to phage T4 in the presence of ephemeral spatial refuges. In the latter environment, selection for genetic resistance is relaxed due to reduced bacteria–phage encounter rates. Moreover, any genetically resistant bacteria cannot spread beyond their hosting spatial refuges in accordance with the predictions generated via our agent-based modelling framework. Finally, it is also conceivable that a minority of bacterial survivors do not encounter phages in the spatial refuges. However, this is unlikely considering that each spatial refuge is explored on average by 24 phages and some of the clones surrounding these phenotypic survivors lyse, releasing around 100 new viral particles per cell.

These novel findings advance our current understanding of resistance to phage, by strengthening recent evidence that in naturally structured environments, the level of genetic resistance evolution is lower compared to within well-mixed environments [26,27,30,33–35,59], possibly because neither phage nor resistant bacteria can freely spread through the population, making the evolution of genetic resistance more costly in natural environments [26]. The evidence above was obtained in heterogeneous spatial environments such as biofilms where bacteria in the colony centre phenotypically survive phage either by reducing their growth or being protected by the biofilm matrix [34,35] and neighbouring cells in the outer rim. Here, we deepen this knowledge by demonstrating, for the first time, that phenotypic but not genetic resistance to lytic phages is an intrinsic fundamental feature of individual bacteria in homogeneous spatial environments where bacteria are isolated from their clonal kin, thus in the absence of population effects such as quorum sensing and biofilm formation. Biofilm formation does not play a role in our structured environments since their surface coating prevents cell attachment; however, it would be interesting in future to contrast the findings reported in this study with measurements performed in spatial refuges permitting bacteria attachment. Quorum sensing involves the production, secretion, and recognition of autoinducers [60] and is likely to occur in our *E*. *coli* BW25113 bulk cultures since the *lsr* operon, responsible for the internalisation and degradation of autoinducers, is significantly up-regulated at the transition from exponential to stationary phase [61], according to previous work on quorum sensing [62]. Quorum sensing can alter the number of phage receptors and phage absorbance [63]. Since it has been shown that OmpC is up-regulated by quorum sensing [64], it is conceivable that at least some *E*. *coli* cells in isolation in spatial refuges down-regulate *ompC* expression. Indeed, we found heterogeneity in *ompC* expression in bacteria isolated in spatial refuges in accordance with previous work [65]. These novel findings reinforce previous work suggesting that in compartmentalised environments with few susceptible hosts, phages tilt their interaction with bacteria towards a mutualistic or prudent use of host resources [66].

One of the historic criticisms of phage therapy is that bacteria readily evolve genetic resistance to phage allowing bacteria to maintain high population densities [19,67]. Here, we show that although phenotypic resistance to phage in structured environments might appear as a bacterial strategy to circumvent phage attack, it does not permit bacteria to maintain high population densities. Our experimental setup is clearly a simplified model system and is not intended to predict phage therapy outcomes but can be used to complement standard microbiological plate and broth culture methods. Nevertheless, our results corroborate previous work suggesting low levels of CRISPR immunity evolution in *Pseudomonas aeruginosa* colonising the lungs (i.e., structured environments) of cystic fibrosis patients [68]. Moreover, low levels of genetic resistance evolution were reported in successful phage therapy case studies in vivo [69–71]. If validated via in vivo model systems, our data will further aid rationally designing phage therapy since we also show that higher phage doses are not always beneficial in clearing bacterial populations in the presence of ephemeral refuges.

Our data also deepen our understanding of the effect of the environment on virulence trade-offs. In well-mixed environments, phages freely propagate among bacteria and rapidly amplify 100-fold in accordance with previous studies [9,20]. In contrast, in the presence of ephemeral refuges, phages need to reach the bacteria-hosting compartments and thereafter amplify only 3-fold. Recent scientific and clinical evidence suggest that in vivo phage therapy often fails due to administration of a lower phage dose than intended [13,69,72,73]. Here, we offer a possible explanation for these failures by demonstrating that the same phage dose amplifies to a lower extent in the presence of ephemeral refuges. We also demonstrate that the phage population is maintained in the absence or presence of spatial refuges despite the emergence of genetic or phenotypic resistance, respectively. These data strengthen the hypothesis that bacterial populations harbour a minority-sensitive population that supports the phage population and that has a competitive advantage over resistant bacteria under conditions of resource limitation [19]. Alternatively, this phage reservoir could be attributed to bacterial subsets that are only transiently phenotypically resistant to phage, a point on which we expand below.

Our data demonstrate that phenotypic resistance to phage is partly due to heterogeneity in the expression of the phage receptor, permitting some bacteria with lower phage receptor abundance to survive phage treatment (OmpC and FadL for phage T4 and T2, respectively). It is conceivable that this differential phage receptor regulation is driven, at least in some individual bacteria, by decreased quorum sensing, which is known to induce *ompC* expression [64], due to bacteria being isolated in spatial refuges. However, cell-to-cell differences in phage receptor expression might not be the only mechanism underlying the observed phenotypic resistance to phage. Phenotypic heterogeneity in the expression of known phage resistance factors could also contribute to the observed phenotypic resistance to phage in structured environments and should be further investigated. For example, differential expression of toxin–antitoxin modules, such as MazEF and ToXI [74,75], or abortive infection systems, such as Rex, Lit, Rnl, or PrrC, could result in the inhibition of phage replication in some cells by inactivating the host transcription and translation machinery [56,76]. Such mechanisms could underlie the nongrowing phenotype we observe that resembles nondividing *E*. *coli* persister cells that are not lysed during phage infection [39] or antibiotic treatment [77] and often rely on dormancy controlled via toxin–antitoxin modules [78]. Phenotypic heterogeneity in the composition of the LPS, another major receptor for T4 [50,79,80], could underlie bacterial survival and duplication in the presence of T4 but in the absence of OmpC. Cell-to-cell differences in the activation of the secondary messenger cGAMP leading to delayed cell lysis [81] could explain our finding that a few bacteria lyse after one or more duplications. Retarded lysis post-phage introduction could also be due to lysis inhibition due to reinfection of a previously infected cell that can prolong the lifetime of the infected bacterium [82,83]. Accordingly, we did not observe phage amplification in the presence of spatial refuges for the treatment with an MOI of 10^*5*^, suggesting that higher phage doses do not necessarily imply greater bacterial clearance in structured environments. Finally, a number of studies have reported bacterial filamentation after prolonged exposure to sublethal stress [84–86] that has been linked to the induction of the SOS response by *recA* in *E*. *coli* [87]. Here, we report, for the first time, that bacteria filamentation occurs also under prolonged exposure to phages, a finding that has remained unrecognised in previous bulk studies. This highlights the importance of investigating bacteria–phage interactions at the single-cell level. Considering that these bacteria typically became longer than their hosting channel by t = 7 h and thus were removed from the mother machine device by the fluid flow in the main channel, we have not pursued this phenotype any further. However, the full characterisation of the fate of filamenting bacteria could be in future carried out by using other microfluidic devices with large and shallow chambers that have been previously employed to characterise bacterial response to antibiotics [85]. This variety of phenotypic responses to phage strengthen the hypothesis that microbial populations can adopt a variety of strategies to survive multiple environmental stressors, thus diversifying the population phenotypic content [88].

In conclusion, by demonstrating that phage-imposed genetic and phenotypic trade-offs are environment dependent and by offering an experimental model system for the investigation of bacteria–phage interactions at the single-cell level, we both deepen the current understanding of the dynamics of phage–bacteria interactions and lay down a stepping stone for dissecting the molecular mechanisms underlying phenotypic survival to phages that plays a crucial role in natural environments.

## Methods

### Materials

All materials were purchased from Fisher Scientific or Sigma-Aldrich unless otherwise stated. LB was purchased from Melford Miller’s and consisted of 10 g/L tryptone, 5 g/L yeast extract, and 10 g/L NaCl. *E*. *coli* strain BW25113, an *ompC* deletion mutant, an *ompC* GFP reporter strain, and a strain harbouring an empty vector, U66, containing GFP but no promoter) were purchased from Dharmacon (GE Healthcare, Lafayette, Colorado, USA). Both the *ompC* reporter strain and the empty vector have a bright, fast-folding GFP with an expressional life span of 8 h in a low-copy plasmid (pMS201) [52]; the promoter region of *ompC* was inserted upstream of GFP in the *ompC* reporter. T2 and T4 phages (ATCC-11303-B2 and ATCC-11303-B4, respectively) were purchased from LGC Standards (LGC Standards, Middlesex, United Kingdom).

### Bacterial culture and phage propagation

Overnight cultures were prepared by picking a single colony of *E*. *coli* BW25113, the *ompC* GFP reporter strain, the empty vector U66, or the *ompC* deletion mutant from an LB agar plate (1.5 g/L agar) and growing it in 200 ml fresh LB on a shaking incubator at 200 rpm and 37°C overnight (i.e., 17 h). Excluding the BW25113 parental strain, the cultures were incubated with 25-μg/ml kanamycin. After 17-h incubation, the culture was diluted 1:1,000 in fresh LB, and growth was measured hourly by taking 3 aliquots that were then centrifuged (13,000 *g* for 5 min), the supernatant was removed, the pellet was resuspended in phosphate-buffered saline (PBS), and serial dilutions were plated on LB agar for colony-forming unit (CFU) counts.

T4 phages were propagated by adding them at an MOI of 1 to a liquid culture of *E*. *coli* BW25113, grown in LB for 6 h, and incubated overnight at 37°C. Phages were collected after centrifugation of the infected overnight culture at 4,000 rpm for 30 min and filtering twice (Medical Millex-GS Filter, 0.22 μm, Millipore). Phages were enumerated via overlay plaque assays and stored at 4°C at a concentration of 5 × 10^*9*^ plaque-forming units (PFUs) ml^*−1*^.

### Infection assay in the unstructured environment

Overnight cultures of *E*. *coli* were diluted in LB so that wells contained varying total numbers of bacteria, from 10^*2*^ up to 10^*4*^ cells per microwell in 96-microwell plates. The loaded dilutions were confirmed through CFU spot assay. After adjusting the phage concentration to achieve an MOI of 1 in every well, 100 μl of bacteria were added to each well along with 100 μl of T4 phage. The plate was incubated at 37°C shaking at 100 rpm, and OD 600 measurements were taken every 30 min for 24 h (CLARIOstar plate reader, BMG LABTECH, Ortenberg, Germany). Calibration curves were used to map OD values to CFUs through periodically sampling the well contents and performing CFU spot assays. The presence of resistant bacteria at 24 h was confirmed through streaking samples onto plates inoculated with phage and incubating over night at 37°C.

Similar infection assays were performed in well-mixed flasks by subculturing overnight *E*. *coli* cultures in flasks containing 100 ml LB at a starting concentration of either 10^*7*^, 10^*6*^, or 10^*4*^ CFUs ml^*−1*^. Phages were added adjusting their concentration to obtain an MOI of either 1, 10^*3*^, or 10^*5*^ depending on the specific experiment. Such flasks were then placed on a shaking incubator at 200 rpm and 37°C for 24 h to maintain an unstructured environment throughout the infection assay. A volume of 20 μl was taken at hourly time points and 10-fold serially diluted in LB down to 10^−8^ for CFU enumeration. A volume of 10 μl of each dilution was spotted on LB agar plates in technical triplicate and left to dry at room temperature. In addition, 200 μl was also taken, 10% chloroform added to each aliquot and each aliquot used for PFU enumeration. In order to do so, each aliquot was lightly vortexed for 10 min, before being centrifuged at 4,000 rpm for 5 min (Eppendorf MiniSpin Microcentrifuge). A volume of 20 μl of the supernatant was taken and 10-fold serially diluted in LB down to 10^−8^. A volume of 5 μl of each dilution were spotted in technical triplicate and incubated overnight at 37°C onto plates prepared as follows: 4 ml of liquid 0.5% LB agar was pipetted onto plates containing 1.5% agar LB followed by 100 μl of an overnight *E*. *coli* culture and left to set at room temperature before spotting phage.

### Infection assay in the structured environment

The microfluidic mother machine device, with ten thousands of compartmentalised channels each hosting between 1 and 6 bacteria, was fabricated and assembled as previously reported [89,90]. Briefly, each channel had a cross section comparable to the size of individual *E*. *coli* cells (≈1 × 1 μm^2^) and a length of 25 μm. These channels were connected to a main microfluidic chamber that was 25 and 100 μm in height and width, respectively. A volume of 10 μl of 0.5 mg/ml bovine serum albumin was added to a 50-ml aliquot of an *E*. *coli* overnight and centrifuged for 5 min at 4,000 rpm and 20°C. The supernatant was filtered twice (Medical Millex-GS Filter, 0.22 μm, Millipore, Burlington, Massachusetts, USA), and 1 ml of this “spent” LB was used to resuspend the bacterial pellet to an OD_600 nm_ (optical density at 600 nm) of 50. This facilitated filling the dead-end channels of the mother machine, while maintaining the bacteria in stationary phase as previously reported [89]. A 2-μl aliquot of the concentrated bacterial suspension above was loaded into the main delivery channel of the mother machine device, and the chip was incubated at 37°C for approximately 20 min. We aimed for approximately 50% of the mother machine dead-end channels to be loaded with 1 or 2 bacteria to facilitate the assessment of the fate of individual bacteria throughout the single-cell infection assay. The chip was then connected to fluorinated ethylene propylene tubing (1/32"  × 0.008"), with the inlet tubing attached to a flow rate unit (Flow Unit S, Fluigent, Paris, France) controlling the pressure applied by a computerised pressure-based flow control system (MFCS-4C, Fluigent), and the outlet tubing into a separate waste tube allowing for the phage-containing outflow to be collected at regular time points.

The chip was mounted on an inverted microscope (IX73 Olympus, Tokyo, Japan) within an environmental chamber kept at 37°C, and spent LB was flushed through the main channel of the chip at 300 μl/h for 8 min before lowering the flow rate to 100 μl/h. A first set of bright field images was acquired via a 60×, 1.2 N.A. objective (UPLSAPO60XW, Olympus) and a sCMOS camera with an exposure time of 0.01 s (Zyla 4.2, Andor, Belfast, United Kingdom). For experiments with the *ompC* GFP reporter strain, we also acquired fluorescence images by using a FITC filter and a blue LED at 20% intensity (CoolLED pE200white, maximal power = 200 mW, Andover, UK). About 50 areas of the mother machine were imaged, each area containing 23 channels, typically hosting 1 or 2 bacteria per channel.

Phages were then diluted to a concentration of 10^*4*^, 10^*7*^, or 10^*9*^ PFU ml^*−1*^ in LB depending on the specific experiment and added to the mother machine at 300 μl/h for 8 min before lowering the flow rate to 100 μl/h. Bright (and fluorescence) images were then acquired every hour for 24 h. At t = 24 h, propidium iodide (PI, Thermo Fisher Scientific, Waltham, Massachusetts, USA) was introduced into the chip to distinguish between viable but nongrowing bacteria (not stained by PI) and dead bacteria with compromised cell walls (stained by PI). Through the chip, 20 μM PI in LB was flowed at 300 μL/h for 8 min then reduced to 100 μl/h for an additional 10 min prior to imaging with a TRITC filter, a green LED at 100% intensity, and a camera exposure time of 0.01 s.

Since each mother machine channel typically accommodates up to 6 bacteria, some of the bacterial progeny was pushed out from the open end of the hosting channels during the single-cell assay, and the fate of such cells during T4 exposure could not be directly measured. Therefore, we predicted the probabilities of the possible fates of these cells (i.e., killed by T4 or surviving T4) based on the measurable fate for the remaining 95% of the population. Moreover, filamentation was defined as a cell over twice the average length of control bacteria growing in the hosting channels of the mother machine (i.e., longer than 7 μm).

In order to measure the phage population dynamics, the mother machine outflow was collected hourly into tubes containing 1 mg/ml kanamycin to prevent phage replication and serially diluted in LB and spotted on plates prepared as follows: 4 ml of liquid 0.5% LB agar was pipetted onto plates containing 1.5% LB agar. A volume of 200 μl of an overnight *E*. *coli* culture (*E*. *coli* BW25113 or *E*. *coli* B in the case of phage T4 and phage T3, respectively) was added to each plate, followed by 100 μL of each phage dilution. Plates were incubated overnight at 37°C, and plaque counts were enumerated. Only plates containing 30 to 300 plaques were counted.

In order to measure the number of particles reaching individual spatial refuges, we used fluorescent nanoparticles with size similar to bacteriophage T4 (i.e., amine-modified polystyrene beads conjugated to orange fluorescent dye, diameter range 100 to 120 nm, Sigma-Aldrich, St. Louis, Missouri, USA). Such nanoparticles were injected in the mother machine at a concentration of 10^*7*^ particles ml^*−1*^ at a flow rate of 100 μl h^−1^, and their diffusion in 20 spatial refuges was continuously imaged for 2 h via a Texas red filter, a green LED at 100% intensity, and a camera exposure time of 0.1 s. This experiment was performed in triplicate.

### Assessment of bacterial genetic resistance to T4

A volume of 100 μl aliquots were taken at hourly time points during infection assays in both the structured and unstructured environments. A volume of 20 μl T4 lysate containing approximately 10^*9*^ PFU ml^*−1*^ was pipetted down an agar plate and let dry. A sterile loop was dipped into each bacterial aliquot and streaked across the plate embedded with T4 phage to verify bacterial resistance to T4.

### Bacterial genome sequencing and variant identification

Aliquots of control bacteria unexposed to T4 phage and bacteria that had become resistant following exposure to T4 for 6 or 24 h in the unstructured environment were grown overnight on agar LB. A single colony was added to 100 μl PBS and streaked onto a second agar plate ensuring 1/3 coverage and incubated overnight at 37°C. All bacteria on the plate were transferred into the barcoded bead tube provided by microbes NG and sent to MicrobesNG for genome sequencing. Aliquots from each overnight culture were washed with extraction buffer containing lysozyme and RNase A and incubated for 25 min at 37°C. Proteinase K and RNaseA were added and incubated for 5 min at 65°C. Genomic DNA was purified using an equal volume of SPRI beads and resuspended in an elution buffer made of 10 mM Tris-HCl (pH 8.5). DNA was quantified in triplicates using the Quantit dsDNA HS assay in an Eppendorff AF2200 plate reader. Genomic DNA libraries were prepared using Nextera XT Library Prep Kit (Illumina, San Diego, United States of America) following the manufacturer’s protocol with the following modifications: 2 ng of DNA were used as input, and the PCR elongation time was 1 min. DNA quantification and library preparation were carried out on a Hamilton Microlab STAR automated liquid handling system. Pooled libraries were quantified using the Kapa Biosystems Library Quantification Kit for Illumina on a Roche light cycler 96 qPCR machine. Libraries were sequenced on the Illumina HiSeq using a 250-bp paired end protocol. Reads were adapter trimmed using Trimmomatic 0.30 with a sliding window quality cutoff of Q15 [91]. De novo assembly was performed on samples using SPAdes version 3.7 [92], and contigs were annotated using Prokka 1.11 [93].

Following filtering, sequence quality scores were assessed with FastQC. As the mean base quality (Phred) scores were >30 for all files, no further filtering was applied. The resultant reads were used to identify mutations against the *E*. *coli* BW25113 reference genome (NCBI reference sequence: NZ_CP009273.1) using the BRESEQ pipeline, default settings, “polymorphism” mode [94]. BRESEQ uses Bowtie2 [95] to map reads to the reference genome then recalibrates base quality scores using base position in the read. SNPs were determined using a negative binomial model to determine SNP likelihood based on read depth coverage. The default threshold frequency of 0.05 was used to identify variants. Deletions were determined from missing coverage and insertions and duplications from read junction evidence. We found no mutations between the reference genome and control bacteria unexposed to T4 phage, therefore did not modify the reference genome prior to mutation calling.

### Image and data analysis

Quantitative information on the bacterial population dynamics in the structured environment was extracted by loading the time-lapse microscopy images of the bacteria-hosting channels in ImageJ. The number of cells present in each channel was counted at each time point, and the cell fate (i.e., live, dead, filamenting, or lost) was assessed using both bright field images and PI staining images at t = 24 h. This analysis was carried out on 200 channels in each experiment allowing to infer the overall bacterial population dynamics in 6,000 channels of each mother machine device. For experiments with the *ompC* reporter strain, the GFP fluorescence levels associated with each bacterium prior to T4 exposure (t = 0) were extracted using the *MMHelper* software [96]. Quantitative information on the diffusion of fluorescent nanoparticles in the structured environment was extracted by loading the time-lapse fluorescence microscopy images in ImageJ and counting the number of particles reaching each spatial refuge during each 2-h long exposure. In order to exclude spurious noise [97,98], we retained as events only those for which a particle explored a spatial refuge for three consecutive frames (i.e., >0.3 s). Particle residence times were measured as the lapse of time between particles entering and leaving the same spatial refuge. Data analysis and plotting was conducted using GraphPad Prism 7. Data were tested for normality using a Shapiro–Wilk normality test. Linear regressions were conducted on the survival fractions from t = 2 h to t = 7 h post-phage addition to extrapolate the intercept to the x-axis, i.e., the predicted time of eradication of the whole bacterial population. Survival fractions at t = 24 h were compared via *t* test.

### Numerical simulations

The mother machine geometry (see Methods section: Infection assay in the structured environment) was simulated as a periodic domain with 1 main and 2 lateral channels, and the Ludwig Lattice–Boltzmann code used to calculate the flow field in this domain [99]. In order to do this, the Navier–Stokes–Fourier equations were solved on a discrete lattice. To reproduce the desired geometry, points of the lattice were marked as either wall or fluid, with each lattice point marking the centre of a cubic element. In this simulation, the side length of each element corresponded to 125 nm. To recreate the pressure gradient acting on the fluid in the main channel of the mother machine, a body force acting along the axis of the main channel was applied uniformly at each lattice point marked as fluid. The Ludwig simulations were run until the flow converged to a steady state. The steady-state flow field was then coupled to an agent-based code written in Python and scaled so that the expected flow rate matched the experimental value of 100 μl h^*−1*^. Phages were modelled as noninteracting point particles following a random walk due to diffusion and advected by the steady-state flow. Phage particles were randomly initialised within the periodic domain, and the time taken for each one to first enter a channel was recorded. The positional accuracy of the simulation was in the order of 100 nm, determined by the expected mean squared displacement of the phage in dimensional units. The timestep in the simulation was chosen to be 5 ms in order to match the positional accuracy above. At this accuracy, data from over 2,600 phages were collected. All simulations were run on a HP EliteOne 800 G3 with Intel Core i5-6500 CPU with 8 GB RAM.

### Agent-based mathematical modelling

We devised a probabilistic individual-based model to investigate the effect of the environmental structure on bacterial population dynamics. The model was constructed to replicate the 2 different experimental setups: (i) unstructured, where bacteria and phage mix freely; and (ii) structured, where the bacteria population is divided into *n* (physically) separated subpopulations in a similar fashion to the bacteria-hosting channels used in the experiments. The initial population size was the same for both setups (i.e., 10^*4*^ bacteria), although the carrying capacity varied (*K*_unstructured_ = 10^*6*^, *K*_structured_ = 4 × 10^*4*^) due to the physical restrictions imposed by the bacteria-hosting channels, where excess bacteria are continuously flushed out of the system. For model simulations, we assumed a maximum capacity of 20 bacteria per bacteria-hosting channels, with *n =* 2,000 bacteria-hosting channels in total.

Bacteria are described by 2 independent resistance-associated traits: (i) surface receptor expression level, ρ; and (ii) genetic resistance, γ. Variation in surface receptor expression is proxied by a phage infection probability ρ (ρ∈[0.5, 1]), with higher expression levels equating to a higher probability that a bacteria–phage encounter leads to infection. Genetic resistance, on the other hand, is a binary trait (γ = 0,1) that is assumed to confer full protection against phage infection—independent of surface receptor expression. Both traits are assumed hereditary, i.e., replicating bacteria pass on their traits to daughter cells albeit with minor variation in receptor expression level (ρ_new_ ~ *Ν*(ρ_old_, 0.1)).

The model is time discrete with a time unit equalling the bacterial generation time. That is, at each timestep, uninfected bacteria double in number up to the carrying capacity. For simplicity, we assumed that cell lysis operates on the same time scale, such that infected bacteria die and release phage from one generation to another. For simplicity, we do not keep track of individual phages released per cell but instead proxy this through the average number of secondary infected bacteria that can arise per infected cell assuming all cells are susceptible. This is given by *R*, which reduces to $R_{t}=RN_{sus}/(N_{sus}+N_{inf})$, with *N*_sus,inf_ being the number of uninfected and infected bacteria, respectively. Note, in the structured environment, there can be significant variation in *R*_t_ between individual channels. Phage infection itself is a probabilistic event, with the total number of possible secondary infections (per channel in the structured environment), *S*_inf_, following a Poisson distribution (*S*_inf_~ Pois(*R*_t_ × *N*_inf_)), and the probability of invasion being successful according to an individual bacteria’s receptor expression level.

We initialised the model with bacteria being at 25% carrying capacity in the structured environment and 1% carrying capacity in the unstructured one (*N*_ini_ = 10^*4*^ for both setups); however, the results presented were independent of the initial population density. We did not account for genetic resistance arising de novo; instead, we assumed a background frequency of resistance of 5/10,000 in accordance with our experimental data. The initial receptor expression level was assumed to be normal with an average of 0.7 and standard deviation SD = 0.08. Due to its probabilistic nature, repeat model runs were necessary to derive robust averages, and the results presented here are based on at least 20 model simulations, seeded with 200 randomly selected bacteria assumed to be infected (as the model does not explicitly account for phages). To illustrate the difference in expression levels between successful and unsuccessful infection events we randomly sampled from 200 potential infections and recorded the bacteria’s surface receptor levels. The model was implemented in C++.

## Supporting information

S1 Table Position, frequency, mutation, annotation, gene, and description of the mutations identified by sequencing the DNA extracted from overnight cultures grown from bacteria that had become resistant to T4 after 6 or 24-h exposure to T4 in the unstructured environment compared to the DNA extracted from control bacteria unexposed to T4 phage. (DOCX)

S1 Fig Temporal dependence of T4 phage density when phages were added at t = 0 to an *E. coli* culture growing exponentially in an unstructured environment (i.e., a flask shaken at 200 rpm and 37°C). Data are the mean and standard error of the mean of measurements performed in biological duplicate and have been normalised to the first reading at t = 6 min. The dashed line is a guide for the eye. Measurements started at 6 min following a 3-min adsorption period and a further 3 min where the sample was diluted 1,000-fold in prewarmed LB broth. Numerical data for each replica are provided in Data A in S1 File. LB, Lysogeny broth; PFU, plaque-forming unit.
(TIFF)

S2 Fig Temporal dependence of bacterial population size in well-mixed 100 μl microwells starting at 3 different bacterial population sizes, 10^*2*^ (black squares), 10^*3*^ (red triangles), and 10^*4*^ (blue circles) bacteria, with T4 phages added at t = 0 at an MOI of 1. Data are the mean and standard error of the mean of 10 biological replicates in 10 microwells. Note that only 10% of the microwells tested displayed regrowth when the initial population was 10^*2*^ bacteria. In contrast, at an initial population of 10^*3*^ and 10^*4*^ bacteria, 40% and 80% of the microwells tested displayed regrowth. Numerical data for each replica are provided in Data C in S1 File. MOI, multiplicity of infection.
(TIFF)

S3 Fig Temporal dependence of bacterial population size in well-mixed 100 ml flasks in the absence (black circles) and presence (red triangles) of T4 phages added at t = 0 at an MOI of 10. Data are the mean and standard error of the mean of biological triplicates. Numerical data for each replica are provided in Data D in S1 File. MOI, multiplicity of infection.
(TIFF)

S4 Fig Temporal dependence of bacterial density when phage T4 were added at t = 0 at an MOI of 1 (circles), 10 (squares), or 10^*3*^ (triangles) to an *E. coli* culture in the unstructured environment (i.e., 100 ml shaken flasks). The phage concentration was kept fixed at 10^*7*^ ml^*−1*^, whereas the bacterial concentration was 10^*7*^, 10^*6*^, or 10^*4*^ ml^*−1*^ to obtain the MOIs indicated above. Data are the mean and standard error of the mean of triplicates. Some of the error bars are hidden behind the corresponding data points. Dashed lines are guides for the eye. Numerical data for each replica are provided in Data E in S1 File. MOI, multiplicity of infection.
(TIFF)

S5 Fig Relative number of individual *E*. *coli* that upon exposure to T4 phage at an MOI of **(A)** 10^*3*^ or **(B)** 10^*5*^ in the structured environment died without duplicating (0), duplicated once (1), twice (2), 3 or more times (3) before death. Data are the mean and standard error of the mean of measurements performed on a total of 450 and 154 *E*. *coli* cells from biological triplicate for MOI 10^*3*^ and 10^*5*^, respectively. Numerical data for each replica are provided in Data G in S1 File. MOI, multiplicity of infection.
(TIFF)

S6 Fig **(A)** Distribution of the number of fluorescent nanoparticles reaching the spatial refuges within 1 h post-addition to the mother machine device. Data and error bars are the mean and standard error of the mean of measurements collected from 60 spatial refuges in 3 different mother machine experiments with 100 nm fluorescent nanoparticles flowing in the device for 2 h. **(B)** Corresponding distribution of residence times (i.e., the lapse of time between the entrance and exit of a particle from a refuge) collated from the 3 experiments above. The dashed line is the distribution median. Numerical data for each replica are provided in Data H and Data I in S1 File, respectively.
(TIFF)

S7 Fig Flow rate dependence of the average number of phages reaching each channel every hour. Data points were obtained via Lattice–Boltzmann simulations, and statistics was collected from over 200 simulated phages in a periodic cross-section of the mother machine. The dashed line is a guide for the eye. Numerical data are provided in Data L in S1 File.
(TIFF)

S8 Fig Temporal dependence of phage density collected in the mother machine outflow when **(A)** T4 phages were added to the structured mother machine environment at t = 0 at a concentration of 10^*7*^ phage ml^*−1*^ in the absence of bacteria and removed from the mother machine environment at t = 2 h, and **(B)** T3 phages were continuously added to the mother machine from t = 0 onwards at a concentration of 10^*7*^ phage ml^*−1*^ in the presence of *E*. *coli* BW25113 (that cannot be infected by phage T3). The dashed lines are guides for the eye. Numerical data for each replica are provided in Data N in S1 File.
(TIFF)

S9 Fig **(A)** Temporal dependence of bacterial population size when phages were continuously injected in the mother machine device at an MOI of 10^*3*^ (triangles) or 10^*5*^ (squares). Data and error bars are the average and standard error of the mean of 530 (triangles) and 208 (squares) single-compartment measurements from biological triplicate experiments in the mother machine. The dashed lines are guides for the eye. Some of the error bars are hidden behind the corresponding data points. **(B)** Corresponding temporal dependence of the concentration of phages collected in the mother machine output. Data are the mean and standard error of the mean of biological triplicates. Dashed lines are guides for the eye. Some of the error bars are hidden behind the corresponding data points. Dotted lines indicate the constant supply of phages in the mother machine input for the 2 MOIs. Data above each respective dotted line indicate phage amplification; data below the corresponding dotted line indicate the absence of phage amplification due to lysis inhibition. Numerical data for each replica are provided in Data J and Data O in S1 File. MOI, multiplicity of infection.
(TIFF)

S10 Fig Representative image of *E. coli* output from the structured environment after 24-h exposure to T4 phage streaked horizontally (from left to right) in triplicate on an LB agar plate. The red arrows indicate T4 phage pipetted along the vertical direction (from top to bottom) on the plate, demonstrating that bacteria did not grow in the presence of phage and hence were not genetically resistant to phage. LB, Lysogeny broth.
(TIF)

S11 Fig Representative fate trajectories of individual *E. coli* cells in ephemeral spatial refuges in the mother machine in the presence of T4 phage from t = 0 onwards. **(A)** A bacterium lysed during or **(B)** after the first hour of exposure to T4 phage. A bacterium duplicated at least once and was killed with all its progeny **(C)** within the first 7 h of the experiment or **(D)** overnight. The dashed lines are guides for the eye. Measurements were carried out in the structured mother machine environment and are representative of *N =* 631 individual bacteria and M = 530 mother machine channels from biological triplicate. Dashed lines are guides for the eye. Numerical values are provided in Data Q in S1 File.
(TIFF)

S12 Fig Temporal dependence of the mean fraction of filamenting bacteria out of a total *N* = 631 *E. coli* in M = 530 mother machine channels from biological triplicate exposed to phage T4. Some of the error bars are hidden behind the corresponding data points due to the large statistical sample. The dashed line is a guide for the eye. Numerical values for each replica are provided in Data R in S1 File.
(TIFF)

S13 Fig Experimental assessment of bacteria viability after 24-h exposure to T4 phage by imaging each bacterium **(A)** in brightfield, **(B)** using GFP fluorescence as a reporter for *ompC* expression, and **(C)** using PI fluorescence. Leftmost channel: representative dead *E*. *coli* cells with compromised membranes stained by PI. Rightmost channel: the first and third *E*. *coli* from the top of the channel are representative viable *E*. *coli* expressing GFP and not stained by PI, the second *E*. *coli* from the top of the channel is a representative dead *E*. *coli* cell with compromised membrane stained by PI. Scale bar: 5 μm. GFP, green fluorescent protein; *ompC*, outer membrane protein C; PI, propidium iodide.
(TIF)

S14 Fig Distributions of GFP levels in *E. coli* harbouring the same GFP-encoding plasmid as the *ompC* and *fadL* reporter strains but without encoding any promoter (i.e., the promoterless plasmid pUA66) before exposure to T4 phage in *E. coli* that later lysed (green violin plot) or survived (black violin plot) exposure to T4. Data were obtained in biological triplicate for a total of *N* = 54, *N* = 17 bacteria that were killed or survived T4 exposure, respectively. Numerical values are provided in Data T in S1 File. GFP, green fluorescent protein; *ompC*, outer membrane protein C.
(TIFF)

S15 Fig Temporal dependence of bacterial population size for the parental (red circles), promoterless plasmid pUA66 (black squares) and *ompC* (blue triangles) reporter strains. Data are the mean and standard error of the mean of biological triplicates in well-mixed 100 ml flasks. Numerical values for each replica are provided in Data U in S1 File. *ompC*, outer membrane protein C.
(TIFF)

S1 File **Data A.** Temporal dependence of phage T4 amplification in the presence of *E*. *coli* BW23113. **Data B.** Temporal dependence of *E*. *coli* BW23113 growth in the presence and absence of phage T4 in well-mixed 200 μl microwells. **Data C.** Temporal dependence of *E*. *coli* BW23113 growth in the presence of phage T4 in well-mixed microwells with different *E*. *coli* inoculum. **Data D.** Temporal dependence of *E*. *coli* BW23113 growth in the presence and absence of phage T4 in well-mixed 100 ml flasks. **Data E.** Temporal dependence of *E*. *coli* BW23113 growth in the presence and absence of phage T4 in well-mixed 100 ml flasks at different MOIs. **Data F.** Temporal dependence of *E*. *coli* BW23113 growth in the presence and absence of phage T4 in the compartments of a microfluidic mother machine. **Data G.** Number of *E*. *coli* generations before lysis in the presence of phage T4 in the compartments of a microfluidic mother machine at a nominal MOI of 10^*3*^ or 10^*5*^. **Data H.** Fractional distribution of the number of nanospheres reaching a compartment of a microfluidic mother machine in 1 h. **Data I.** Fractional distribution of the residence time of nanospheres in the compartments of a microfluidic mother machine. **Data J.** Temporal dependence of *E*. *coli* BW23113 growth in the presence of phage T4 at 3 different MOIs in the compartments of a microfluidic mother machine. **Data K.** Temporal dependence of the simulated fraction of mother machine compartments that have not been reached by phage. **Data L.** Flow rate dependence of the number of phage reaching each compartment of a microfluidic mother machine. **Data M.** Temporal dependence of phage T4 amplification in the presence of *E*. *coli* BW23113 in either well-mixed 100 ml flasks or the compartments of a microfluidic mother machine. **Data N.** Temporal dependence of either phage T4 or phage T3 amplification in the absence or presence of *E*. *coli* BW23113, respectively, in the compartments of a microfluidic mother machine. **Data O.** Temporal dependence of phage T4 amplification in the presence of *E*. *coli* BW23113 at 2 different MOIs in the compartments of a microfluidic mother machine. **Data P.** Representative temporal dependence of single *E*. *coli* BW23113 surviving in the presence of phage T4 in the compartments of a microfluidic mother machine. **Data Q.** Representative temporal dependence of single *E*. *coli* BW23113 lysing in the presence of phage T4 in the compartments of a microfluidic mother machine. **Data R.** Temporal dependence of the fraction of filamenting *E*. *coli* BW23113 in the presence of phage T4 in the compartments of a microfluidic mother machine. **Data S.** Distribution of single-cell GFP fluorescence reporting on the expression of either *ompC* or *fadL* for *E*. *coli* BW23113 susceptible of surviving phage T4 or phage T2. **Data T.** Distribution of single-cell GFP fluorescence for a promoterless *E*. *coli* BW23113 strain susceptible of surviving phage T4. **Data U.** Temporal dependence of the growth of either the parental *E*. *coli* BW23113 strain or the *ompC* reporter strain or the promoterless strain in 100 ml flasks. **Data V.** Temporal dependence of the growth of either the parental *E*. *coli* BW23113 strain or the Δ*ompC* deletion mutant in the presence of phage T4 in the compartments of a microfluidic mother machine. **Data W.** Distribution of the number of bacterial duplications before death for either the parental *E*. *coli* BW23113 strain or the Δ*ompC* deletion mutant in the presence of phage T4 in the compartments of a microfluidic mother machine. **Data X.** Temporal dependence of bacterial growth, genetic or phenotypic resistance to phage either in an unstructured or a structured environment according to agent-based simulations. **Data Y.** Distribution of single-cell phage receptor expression in bacteria that are either susceptible or survive phage exposure in the structured environment according to agent-based simulations. GFP, green fluorescent protein; MOI, multiplicity of infection; *ompC*, outer membrane protein C.
(XLSX)

## Abbreviations

CFU: colony-forming unit
GFP: green fluorescent protein
LB: Lysogeny broth
LPS: lipopolysaccharide
MOI: multiplicity of infection
OmpC: outer membrane protein C
PBS: phosphate-buffered saline
PFU: plaque-forming unit
PI: propidium iodide
SNP: single nucleotide polymorphism
