# Supplementary material for: Individual bacteria in structured environments rely on phenotypic resistance to phage
Source: PLoS Biol. 2021 Oct 12;19(10):e3001406. doi: 10.1371/journal.pbio.3001406 (PMC8509860; doi:10.1371/journal.pbio.3001406)
Supplement: S1 Table — (DOCX) [file pbio.3001406.s001.docx]

| **Time (h)** | **Position** | **Frequency** | **Mutation** | **Annotation** | **Gene** | **Description** |
| --- | --- | --- | --- | --- | --- | --- |
| 6 | 360,104 | 51.8% | 183 bp x 2 | duplication | *lacZ* ← / ← *lacI* | pseudogene, truncated/DNA‑binding transcriptional repressor |
| 6 | 501,107 | 5.3% | G→A | A246A (GCG→GCA) | *ushA* → | bifunctional UDP‑sugar hydrolase/5'‑nucleotidase |
| 6 | 1,266,001 | 6.8% | G→T | intergenic (+85/+204) | *rdlC* → / ← *chaA* | sRNA antisense regulator affects LdrC translation; proposed addiction module in LDR‑C repeat, with toxic peptide LdrC/calcium/sodium:proton antiporter |
| 6 | 1,460,458 | 13.6% | T→C | pseudogene (810/2513 nt) | *ydbA* → | pseudogene, autotransporter homolog; interrupted by IS2 and IS30 |
| 6 | 1,460,467 | 11.4% | G→A | pseudogene (819/2513 nt) | *ydbA* → | pseudogene, autotransporter homolog; interrupted by IS2 and IS30 |
| **6** | **2,017,304** | **100%** | **C→T** | **intergenic (+145/‑145)** | ***fliR* → / → *rcsA*** | **flagellar export pore protein/transcriptional regulator of colanic acid capsular biosynthesis** |
| **6** | **2,309,836** | **100%** | **G→A** | **G61S (GGC→AGC)** | ***rcsB* →** | **response regulator in two‑component regulatory system with RcsC and YojN** |
| **6** | **2,670,665** | **100%** | **A→T** | **I352I (ATT→ATA)** | ***yphE* ←** | **putative sugar transporter subunit of ABC superfamily, ATP‑binding component** |
| **6** | **3,521,110** | **100%** | **T→A** | **I428N (ATC→AAC)** | ***yrfF* →** | **inner membrane protein** |
| 6 | 4,511,057 | 5.2% | A→G | L261L (TTA→CTA) | *yjhF* ← | putative transporter |
| 24 | 1,264,818 | 7.1% | G→T | noncoding (40/67 nt) | *rdlA* → | sRNA antisense regulator affects LdrA translation; proposed addiction module in LDR‑A repeat, with toxic peptide LdrA |
| 24 | 1,265,182 | 7.6% | C→T | G29S (GGC→AGC) | *ldrB* ← | toxic polypeptide, small |
| 24 | 1,355,081 | 6.6% | G→T | R29S (CGC→AGC) | *puuA* ← | glutamate‑‑putrescine ligase |
| 24 | 1,460,458 | 9.5% | T→C | pseudogene (810/2513 nt) | *ydbA* → | pseudogene, autotransporter homolog; interrupted by IS2 and IS30 |
| 24 | 1,460,467 | 7.8% | G→A | pseudogene (819/2513 nt) | *ydbA* → | pseudogene, autotransporter homolog; interrupted by IS2 and IS30 |
| 24 | 1,703,187 | 7.9% | C→A | P671Q (CCG→CAG) | *rsxC* → | SoxR iron‑sulfur cluster reduction factor component; putative membrane‑associated NADH oxidoreductase of electron transport complex |
| **24** | **2,017,304** | **100%** | **C→T** | **intergenic (+145/‑145)** | ***fliR* → / → *rcsA*** | **flagellar export pore protein/transcriptional regulator of colanic acid capsular biosynthesis** |
| 24 | 2,962,056 | 6.2% | C→T | G94D (GGC→GAC) | *rppH* ← | RNA pyrophosphohydrolase |
