# Supplementary figures and images for: Individual bacteria in structured environments rely on phenotypic resistance to phage
Source: PLoS Biol. 2021 Oct 12;19(10):e3001406. doi: 10.1371/journal.pbio.3001406 (PMC8509860; doi:10.1371/journal.pbio.3001406)

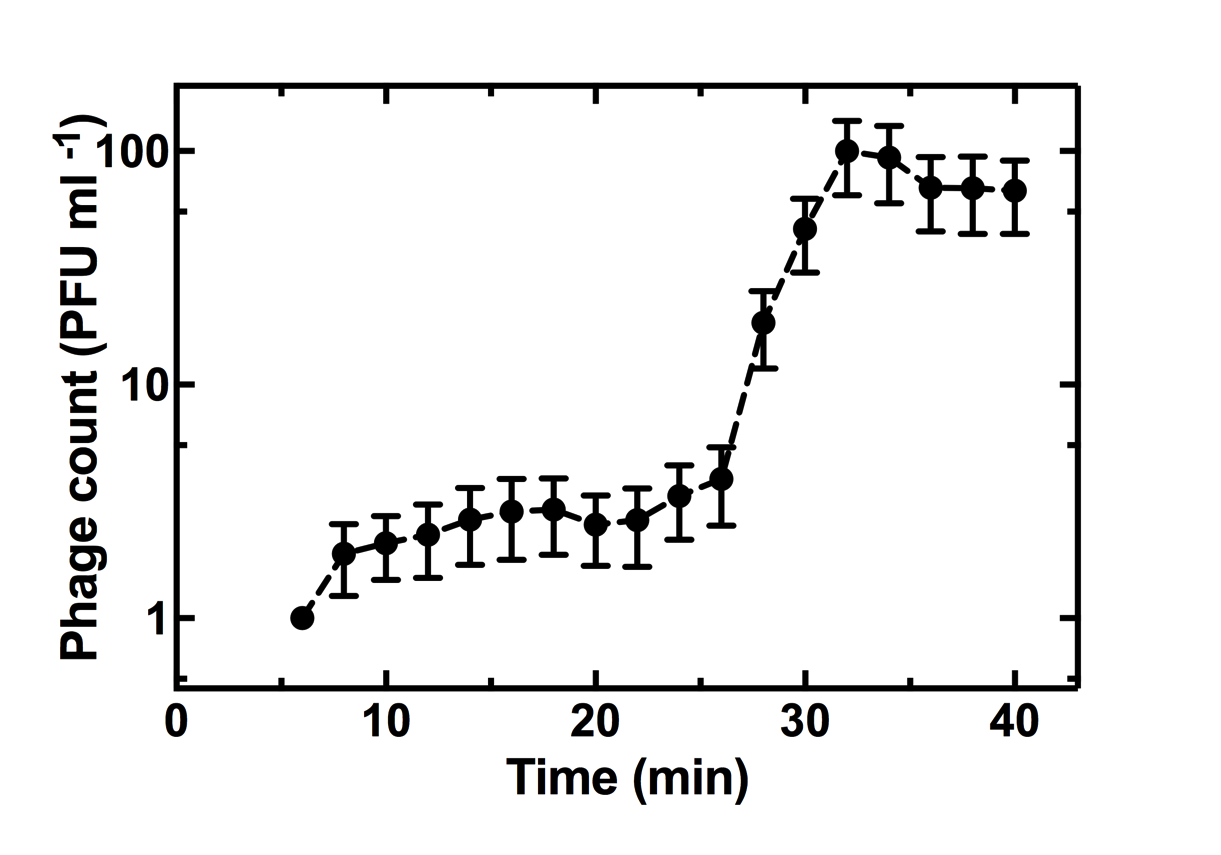

Supplement: S1 Fig — Data are the mean and standard error of the mean of measurements performed in biological duplicate and have been normalised to the first reading at t = 6 min. The dashed line is a guide for the eye. Measurements started at 6 min following a 3-min adsorption period and a further 3 min where the sample was diluted 1,000-fold in prewarmed LB broth. Numerical data for each replica are provided in Data A in S1 File. LB, Lysogeny broth; PFU, plaque-forming unit. (TIFF) [file pbio.3001406.s002.tiff]

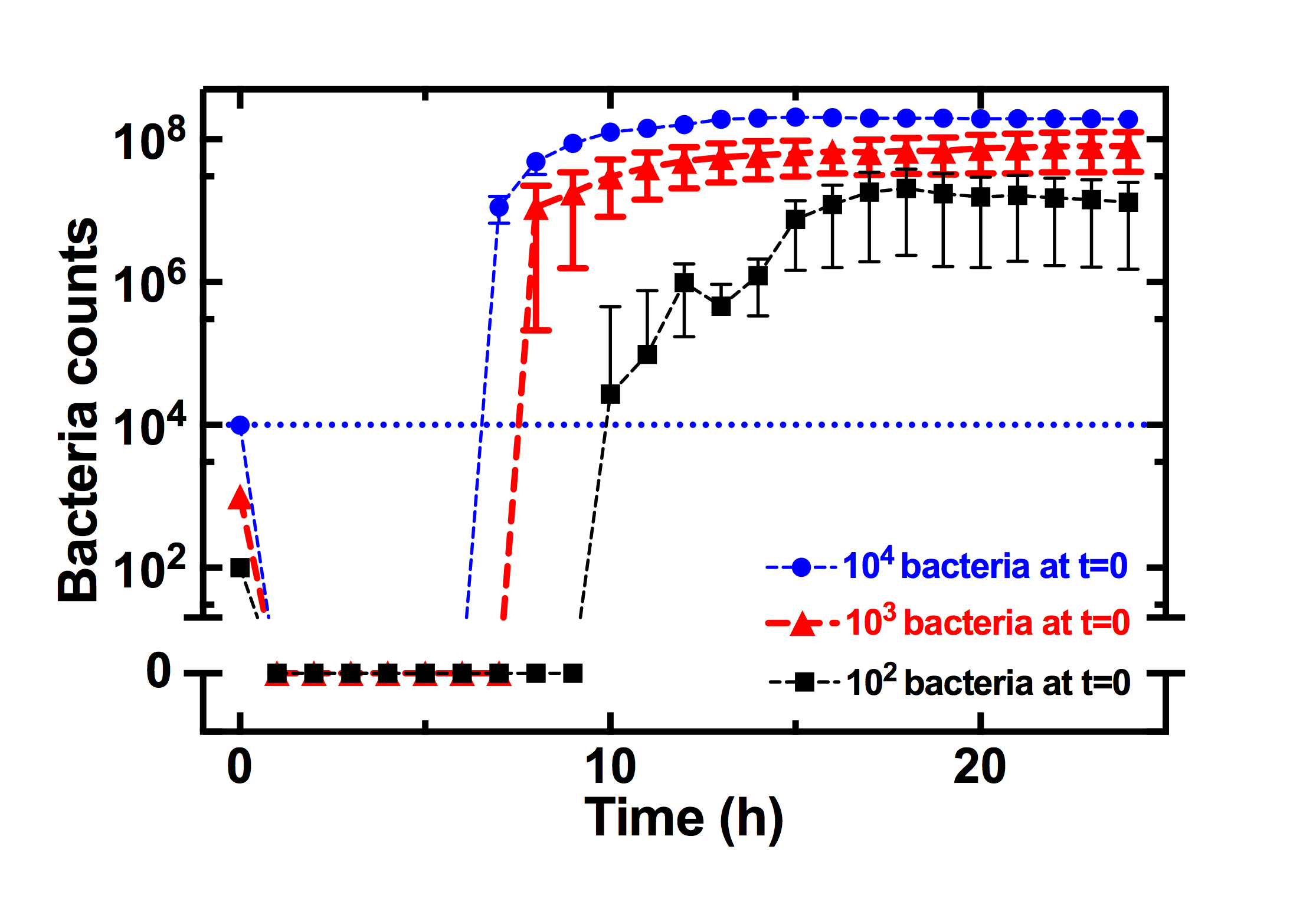

Supplement: S2 Fig — Data are the mean and standard error of the mean of 10 biological replicates in 10 microwells. Note that only 10% of the microwells tested displayed regrowth when the initial population was 102 bacteria. In contrast, at an initial population of 103 and 104 bacteria, 40% and 80% of the microwells tested displayed regrowth. Numerical data for each replica are provided in Data C in S1 File. MOI, multiplicity of infection. (TIFF) [file pbio.3001406.s003.tiff]

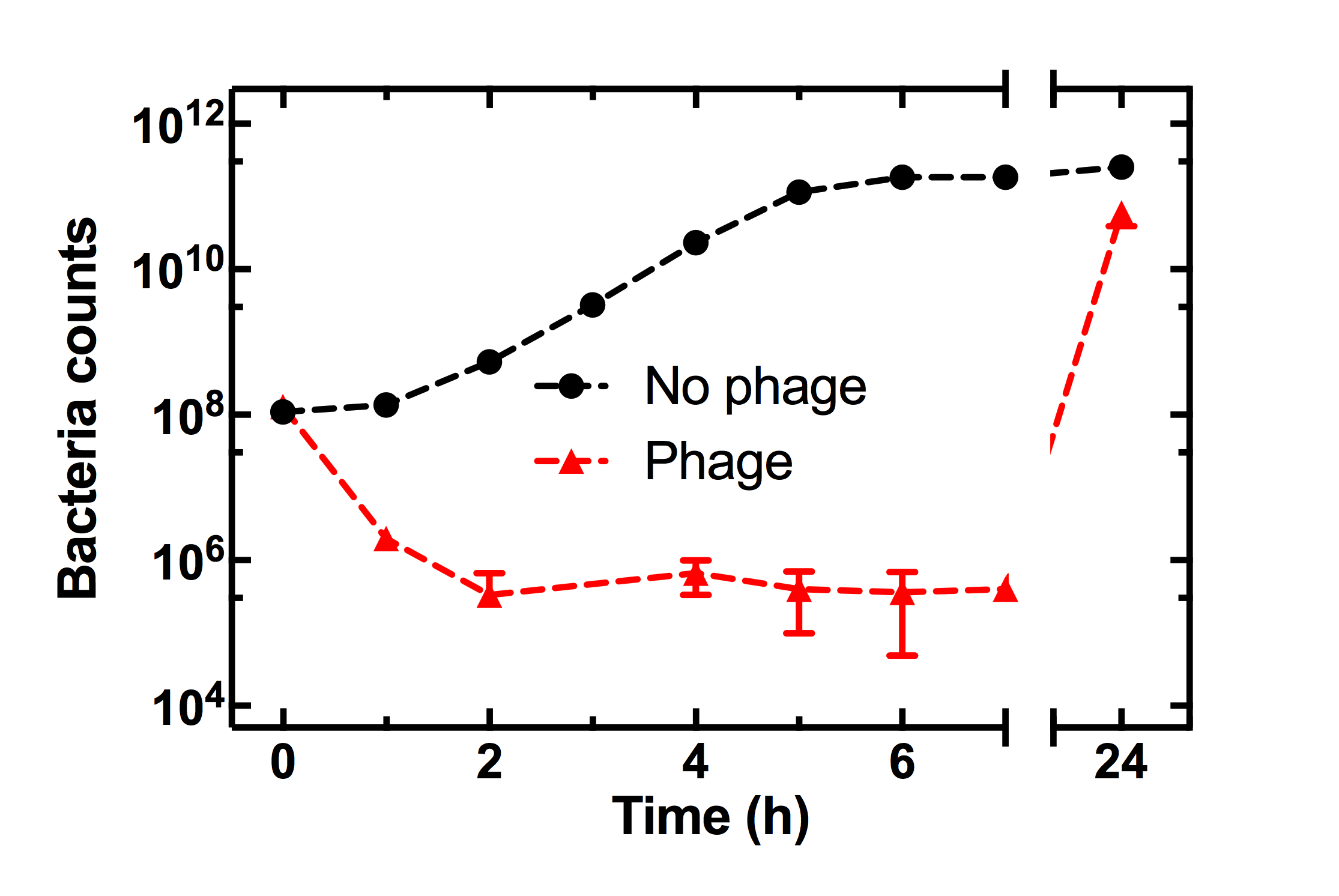

Supplement: S3 Fig — Data are the mean and standard error of the mean of biological triplicates. Numerical data for each replica are provided in Data D in S1 File. MOI, multiplicity of infection. (TIFF) [file pbio.3001406.s004.tiff]

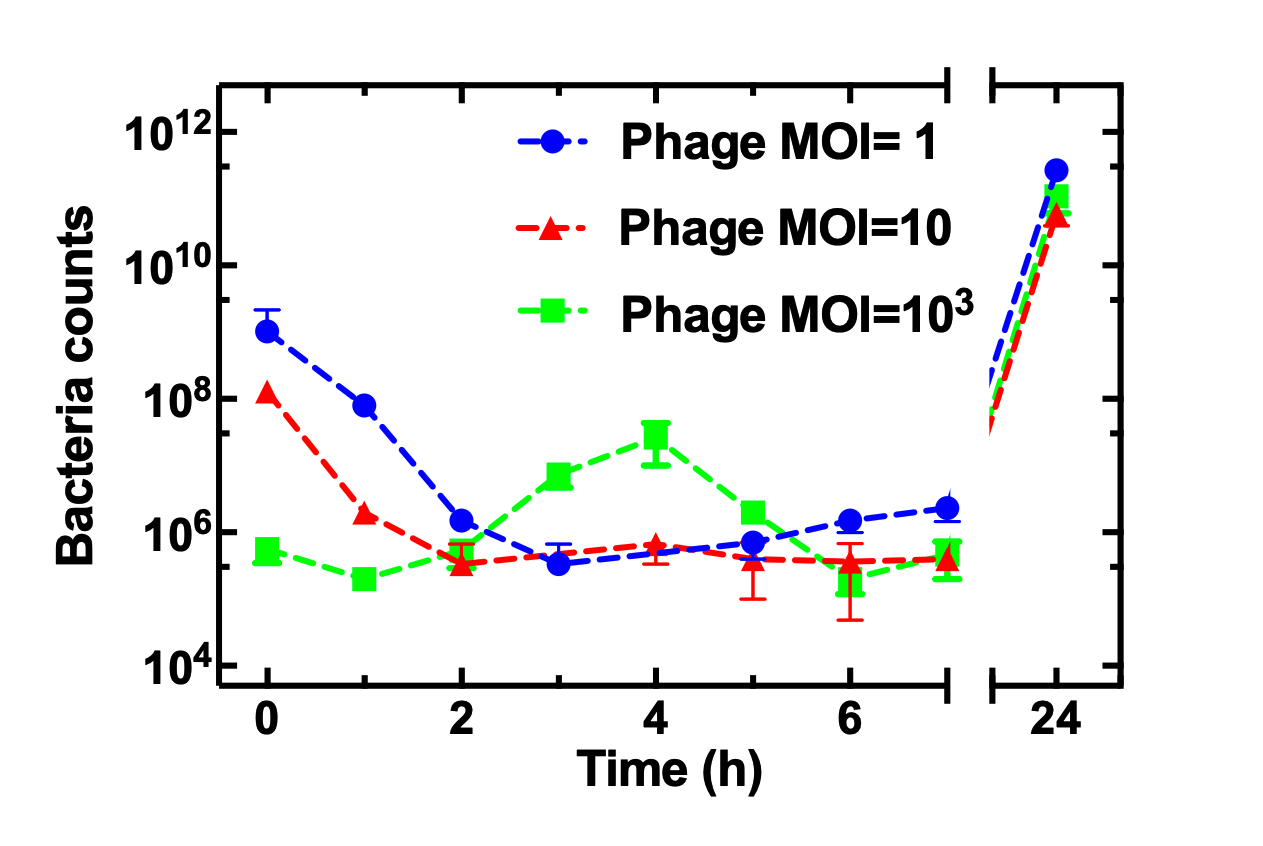

Supplement: S4 Fig — The phage concentration was kept fixed at 107 ml−1, whereas the bacterial concentration was 107, 106, or 104 ml−1 to obtain the MOIs indicated above. Data are the mean and standard error of the mean of triplicates. Some of the error bars are hidden behind the corresponding data points. Dashed lines are guides for the eye. Numerical data for each replica are provided in Data E in S1 File. MOI, multiplicity of infection. (TIFF) [file pbio.3001406.s005.tiff]

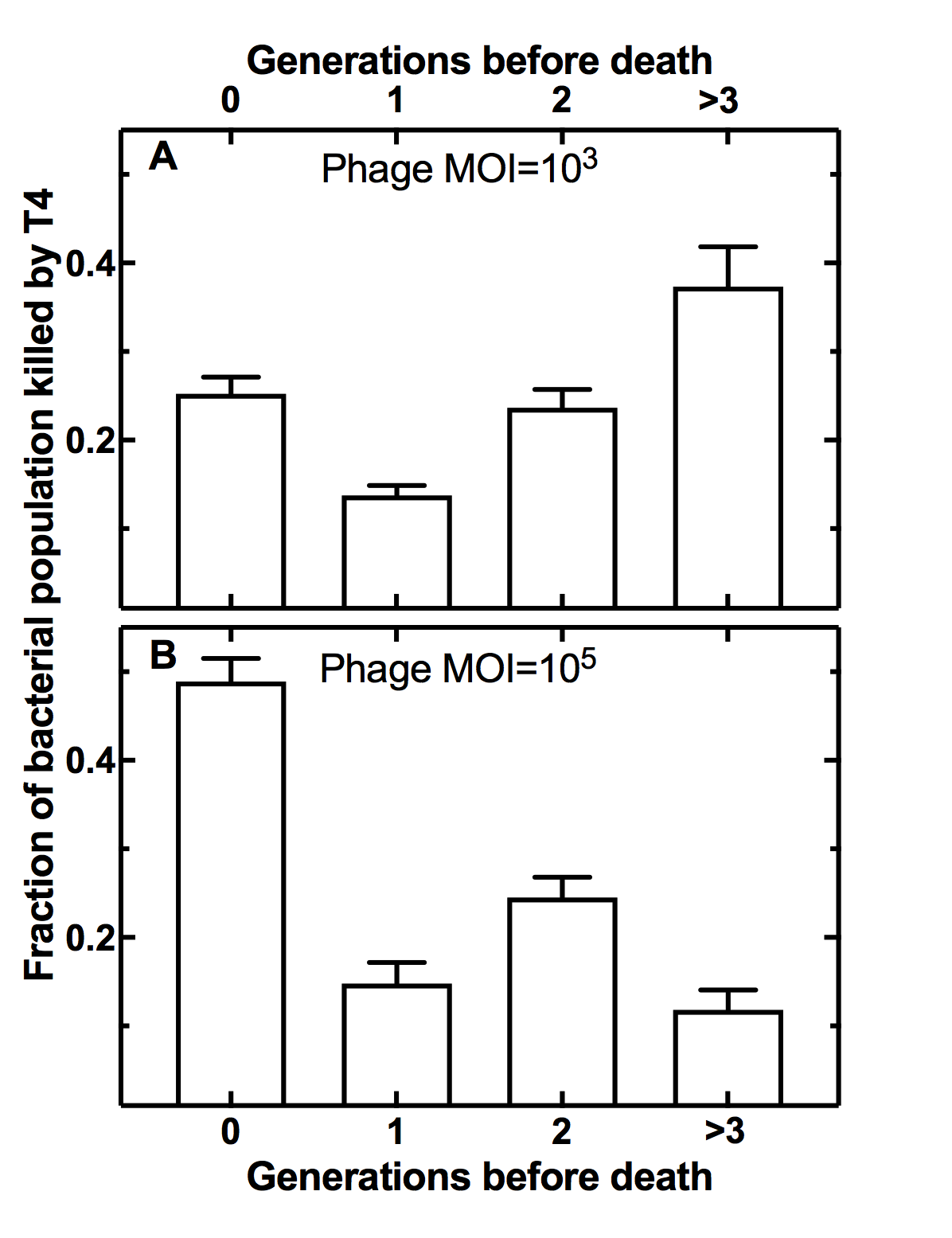

Supplement: S5 Fig — Relative number of individual E. coli that upon exposure to T4 phage at an MOI of (A) 103 or (B) 105 in the structured environment died without duplicating (0), duplicated once (1), twice (2), 3 or more times (3) before death. Data are the mean and standard error of the mean of measurements performed on a total of 450 and 154 E. coli cells from biological triplicate for MOI 103 and 105, respectively. Numerical data for each replica are provided in Data G in S1 File. MOI, multiplicity of infection. (TIFF) [file pbio.3001406.s006.tiff]

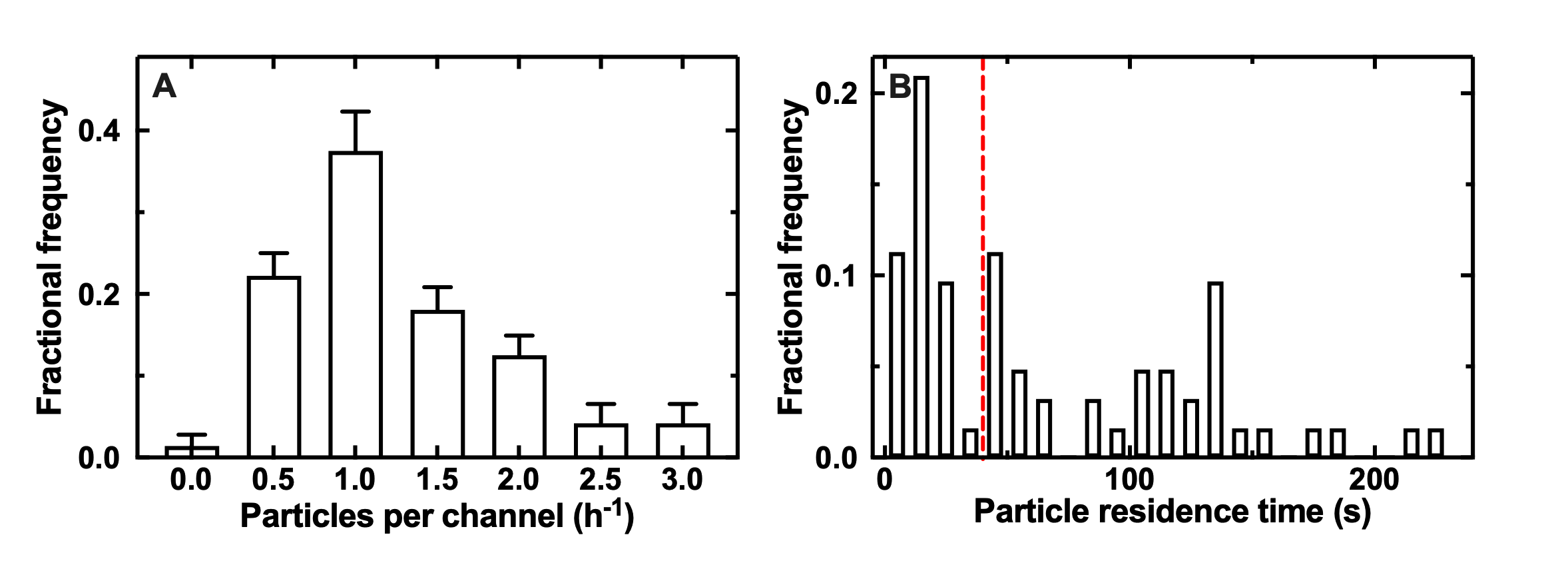

Supplement: S6 Fig — (A) Distribution of the number of fluorescent nanoparticles reaching the spatial refuges within 1 h post-addition to the mother machine device. Data and error bars are the mean and standard error of the mean of measurements collected from 60 spatial refuges in 3 different mother machine experiments with 100 nm fluorescent nanoparticles flowing in the device for 2 h. (B) Corresponding distribution of residence times (i.e., the lapse of time between the entrance and exit of a particle from a refuge) collated from the 3 experiments above. The dashed line is the distribution median. Numerical data for each replica are provided in Data H and Data I in S1 File, respectively. (TIFF) [file pbio.3001406.s007.tiff]

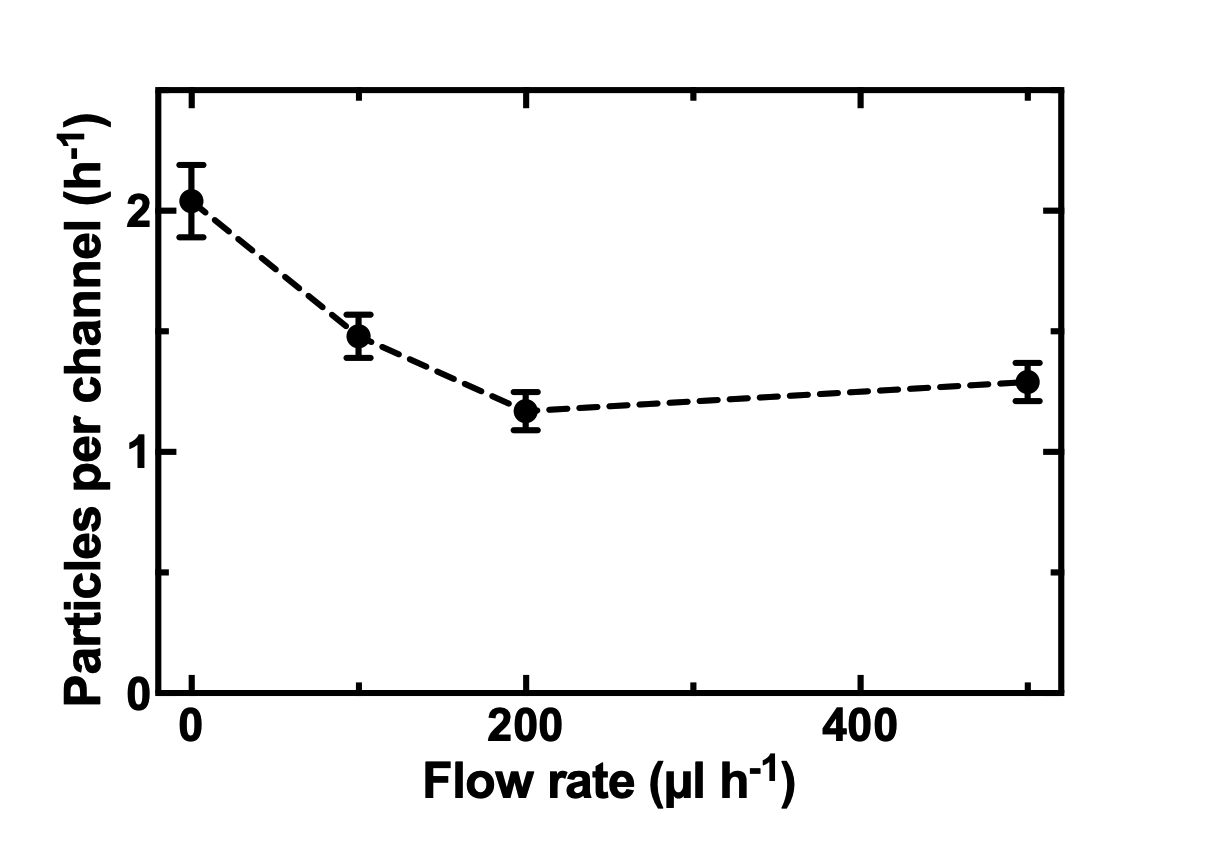

Supplement: S7 Fig — Data points were obtained via Lattice–Boltzmann simulations, and statistics was collected from over 200 simulated phages in a periodic cross-section of the mother machine. The dashed line is a guide for the eye. Numerical data are provided in Data L in S1 File. (TIFF) [file pbio.3001406.s008.tiff]

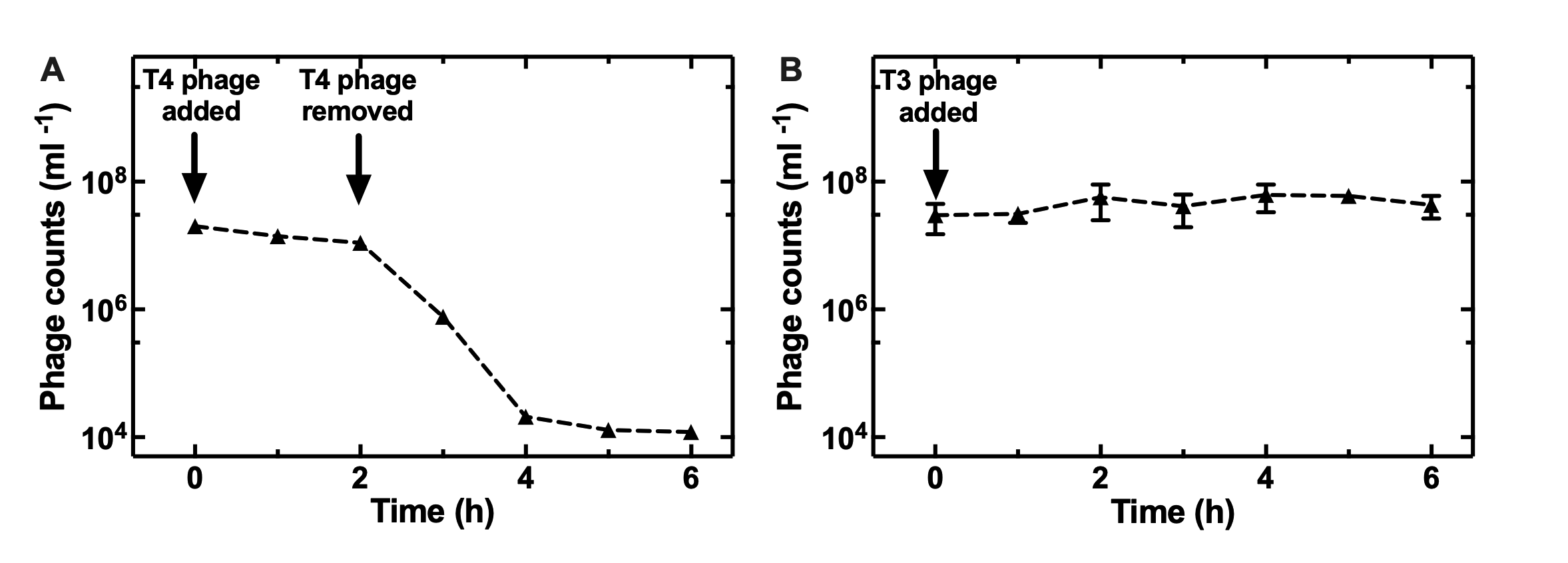

Supplement: S8 Fig — Temporal dependence of phage density collected in the mother machine outflow when (A) T4 phages were added to the structured mother machine environment at t = 0 at a concentration of 107 phage ml−1 in the absence of bacteria and removed from the mother machine environment at t = 2 h, and (B) T3 phages were continuously added to the mother machine from t = 0 onwards at a concentration of 107 phage ml−1 in the presence of E. coli BW25113 (that cannot be infected by phage T3). The dashed lines are guides for the eye. Numerical data for each replica are provided in Data N in S1 File. (TIFF) [file pbio.3001406.s009.tiff]

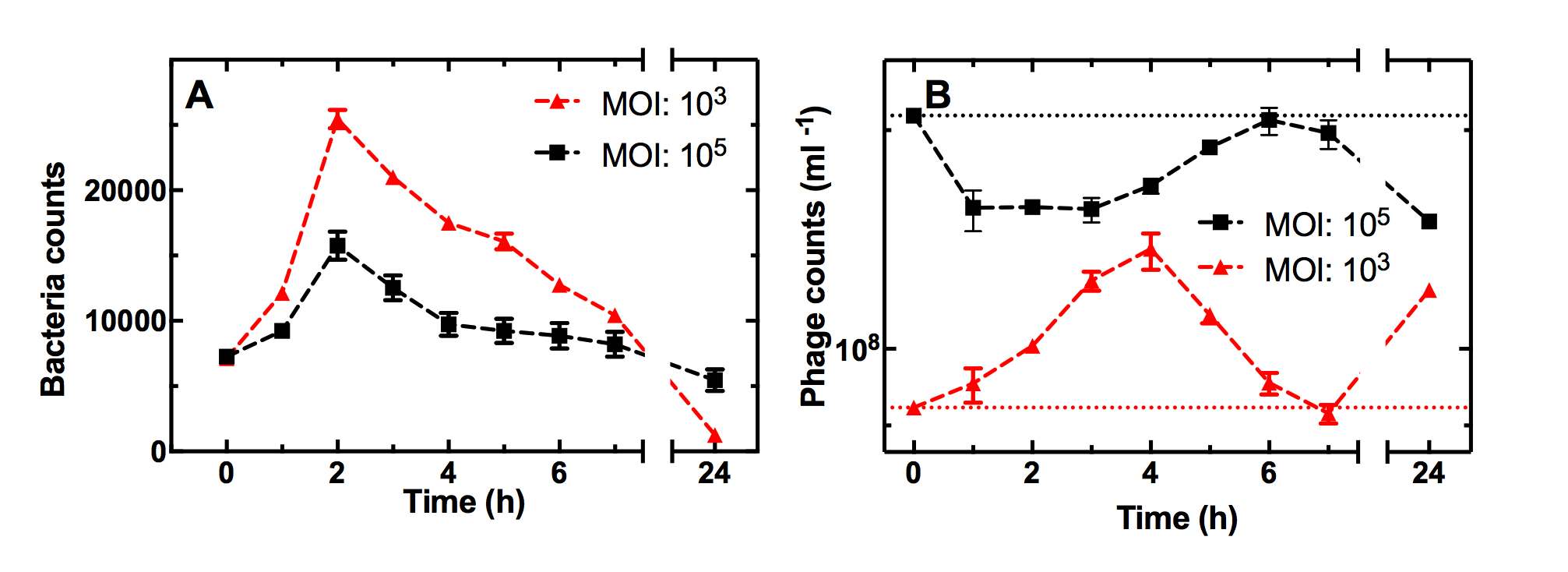

Supplement: S9 Fig — (A) Temporal dependence of bacterial population size when phages were continuously injected in the mother machine device at an MOI of 103 (triangles) or 105 (squares). Data and error bars are the average and standard error of the mean of 530 (triangles) and 208 (squares) single-compartment measurements from biological triplicate experiments in the mother machine. The dashed lines are guides for the eye. Some of the error bars are hidden behind the corresponding data points. (B) Corresponding temporal dependence of the concentration of phages collected in the mother machine output. Data are the mean and standard error of the mean of biological triplicates. Dashed lines are guides for the eye. Some of the error bars are hidden behind the corresponding data points. Dotted lines indicate the constant supply of phages in the mother machine input for the 2 MOIs. Data above each respective dotted line indicate phage amplification; data below the corresponding dotted line indicate the absence of phage amplification due to lysis inhibition. Numerical data for each replica are provided in Data J and Data O in S1 File. MOI, multiplicity of infection. (TIFF) [file pbio.3001406.s010.tiff]

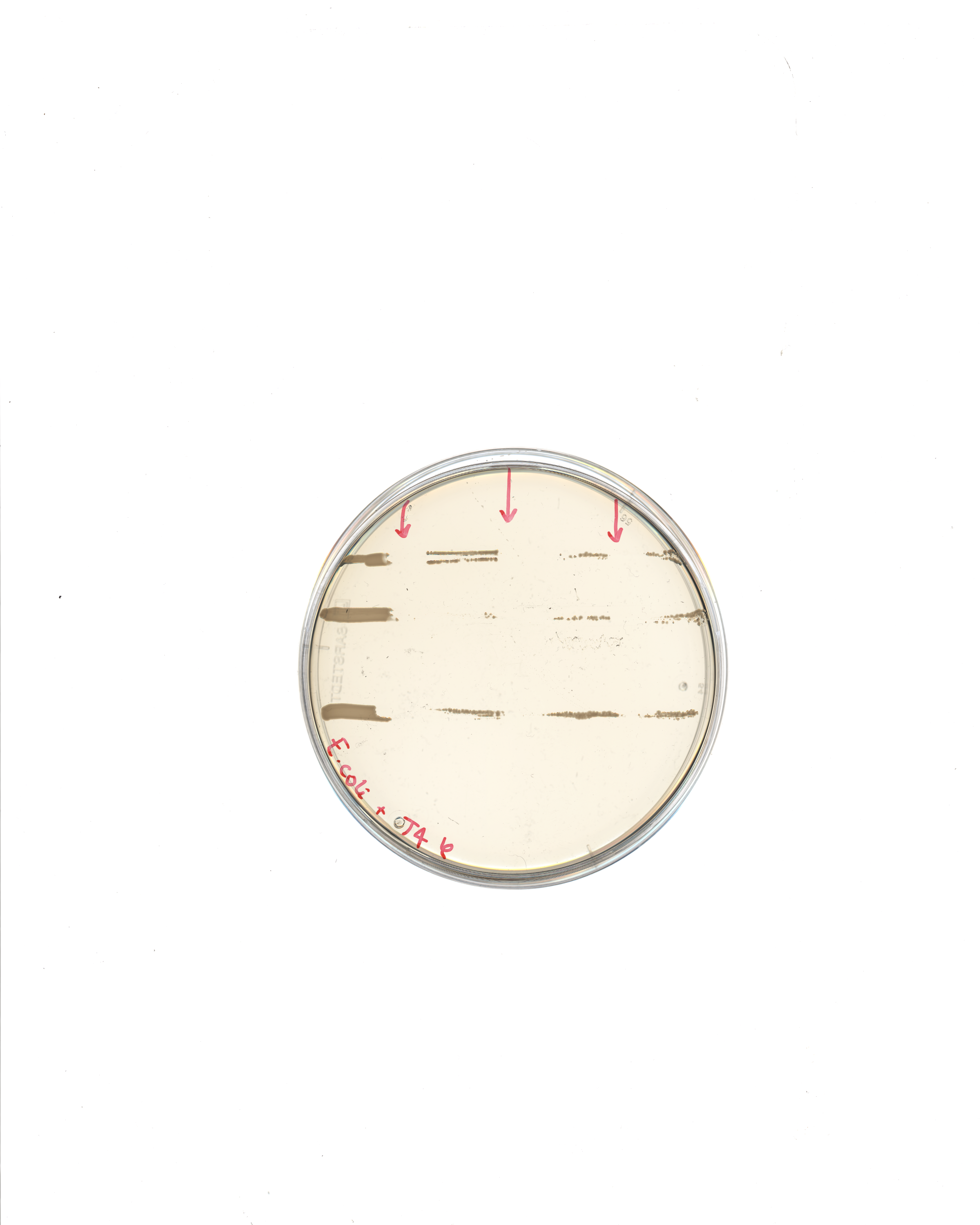

Supplement: S10 Fig — The red arrows indicate T4 phage pipetted along the vertical direction (from top to bottom) on the plate, demonstrating that bacteria did not grow in the presence of phage and hence were not genetically resistant to phage. LB, Lysogeny broth. (TIF) [file pbio.3001406.s011.tif]

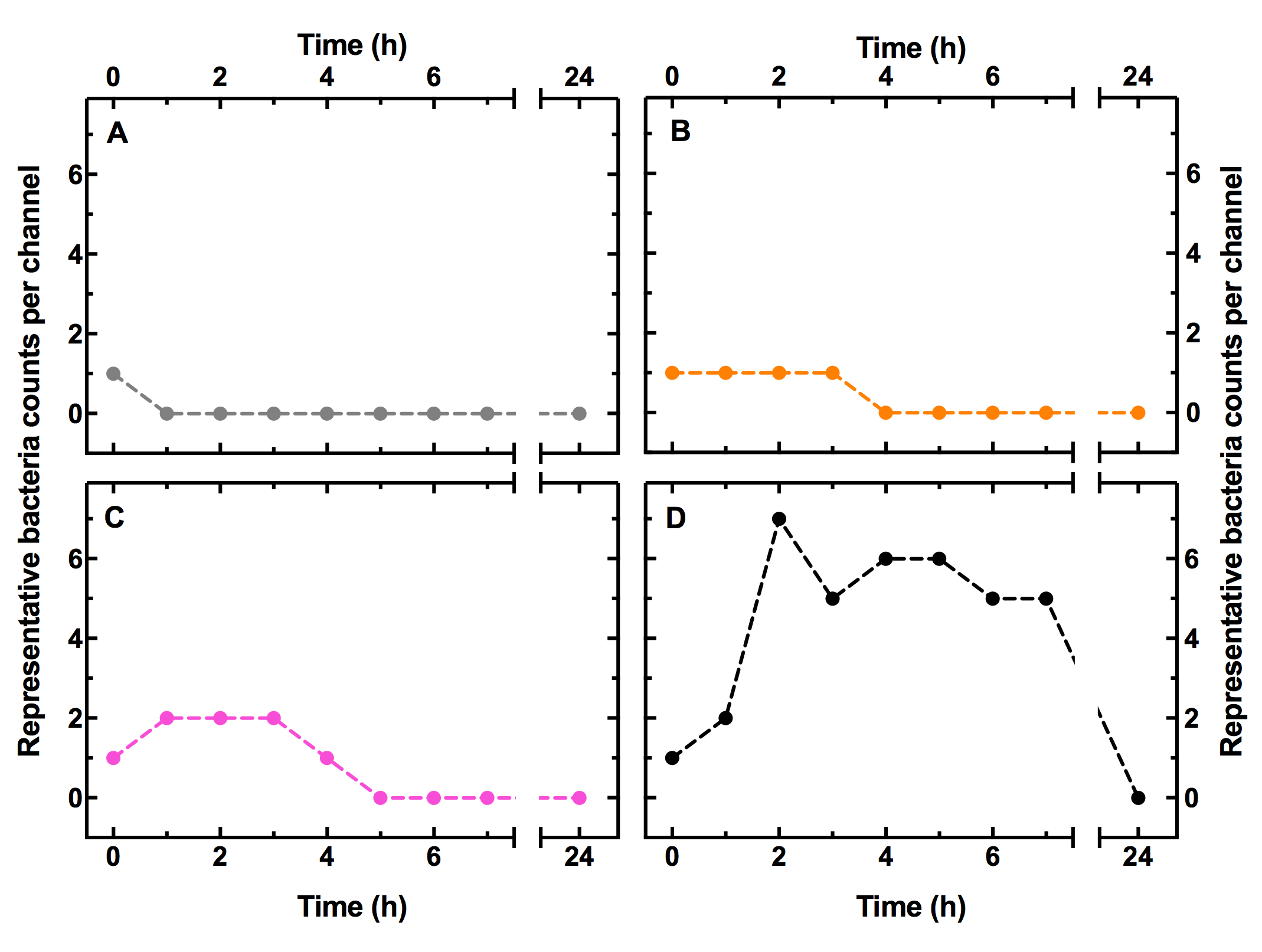

Supplement: S11 Fig — (A) A bacterium lysed during or (B) after the first hour of exposure to T4 phage. A bacterium duplicated at least once and was killed with all its progeny (C) within the first 7 h of the experiment or (D) overnight. The dashed lines are guides for the eye. Measurements were carried out in the structured mother machine environment and are representative of N = 631 individual bacteria and M = 530 mother machine channels from biological triplicate. Dashed lines are guides for the eye. Numerical values are provided in Data Q in S1 File. (TIFF) [file pbio.3001406.s012.tiff]

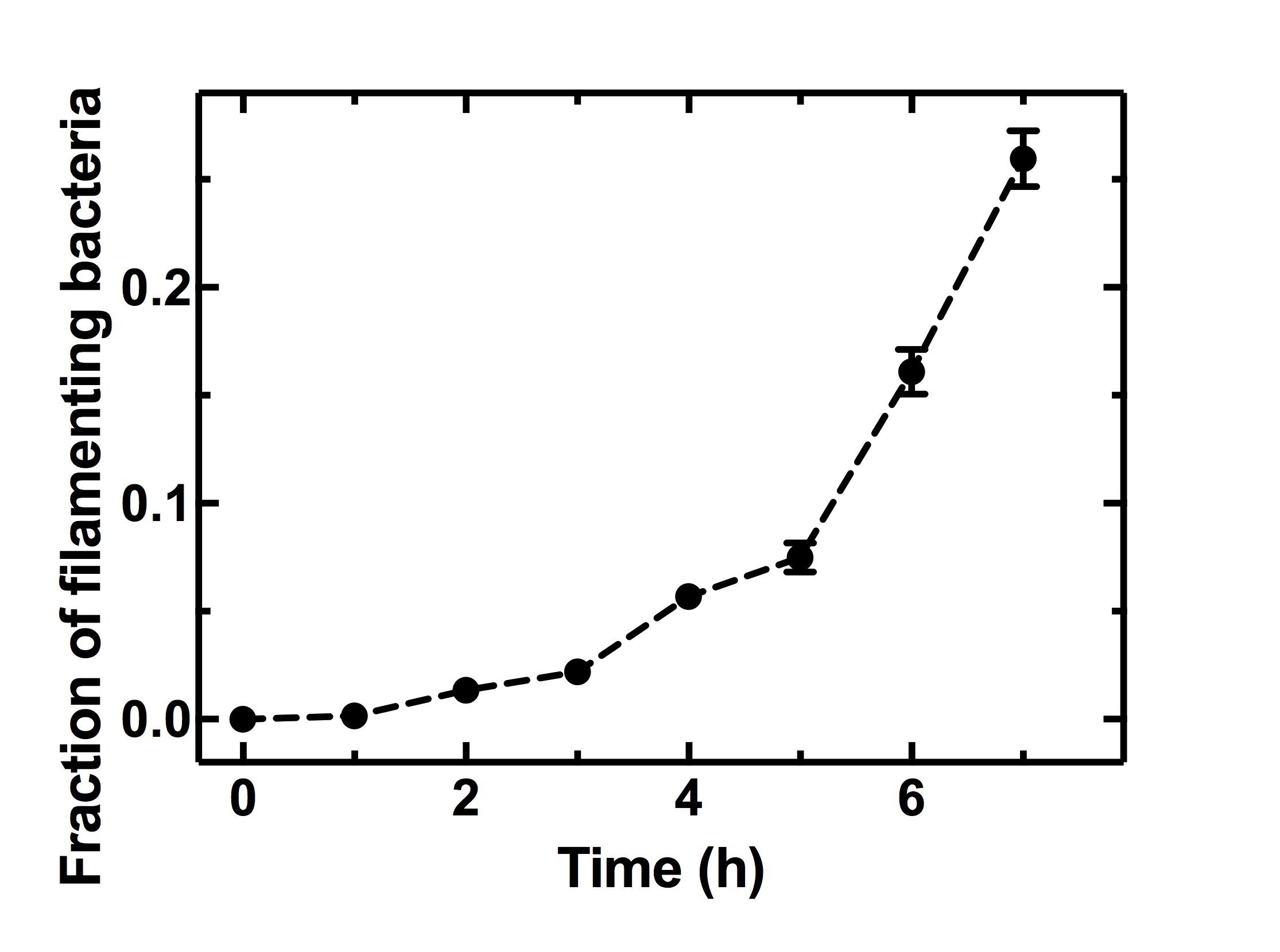

Supplement: S12 Fig — Some of the error bars are hidden behind the corresponding data points due to the large statistical sample. The dashed line is a guide for the eye. Numerical values for each replica are provided in Data R in S1 File. (TIFF) [file pbio.3001406.s013.tiff]

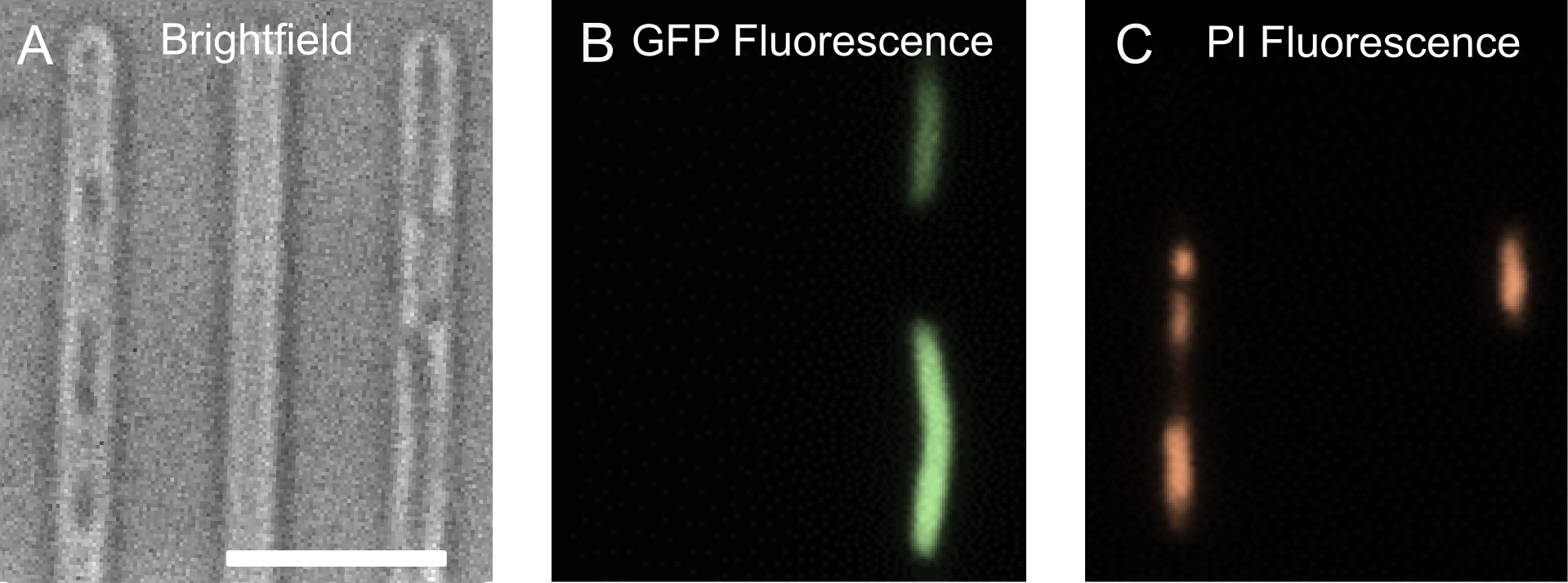

Supplement: S13 Fig — Experimental assessment of bacteria viability after 24-h exposure to T4 phage by imaging each bacterium (A) in brightfield, (B) using GFP fluorescence as a reporter for ompC expression, and (C) using PI fluorescence. Leftmost channel: representative dead E. coli cells with compromised membranes stained by PI. Rightmost channel: the first and third E. coli from the top of the channel are representative viable E. coli expressing GFP and not stained by PI, the second E. coli from the top of the channel is a representative dead E. coli cell with compromised membrane stained by PI. Scale bar: 5 μm. GFP, green fluorescent protein; ompC, outer membrane protein C; PI, propidium iodide. (TIF) [file pbio.3001406.s014.tif]

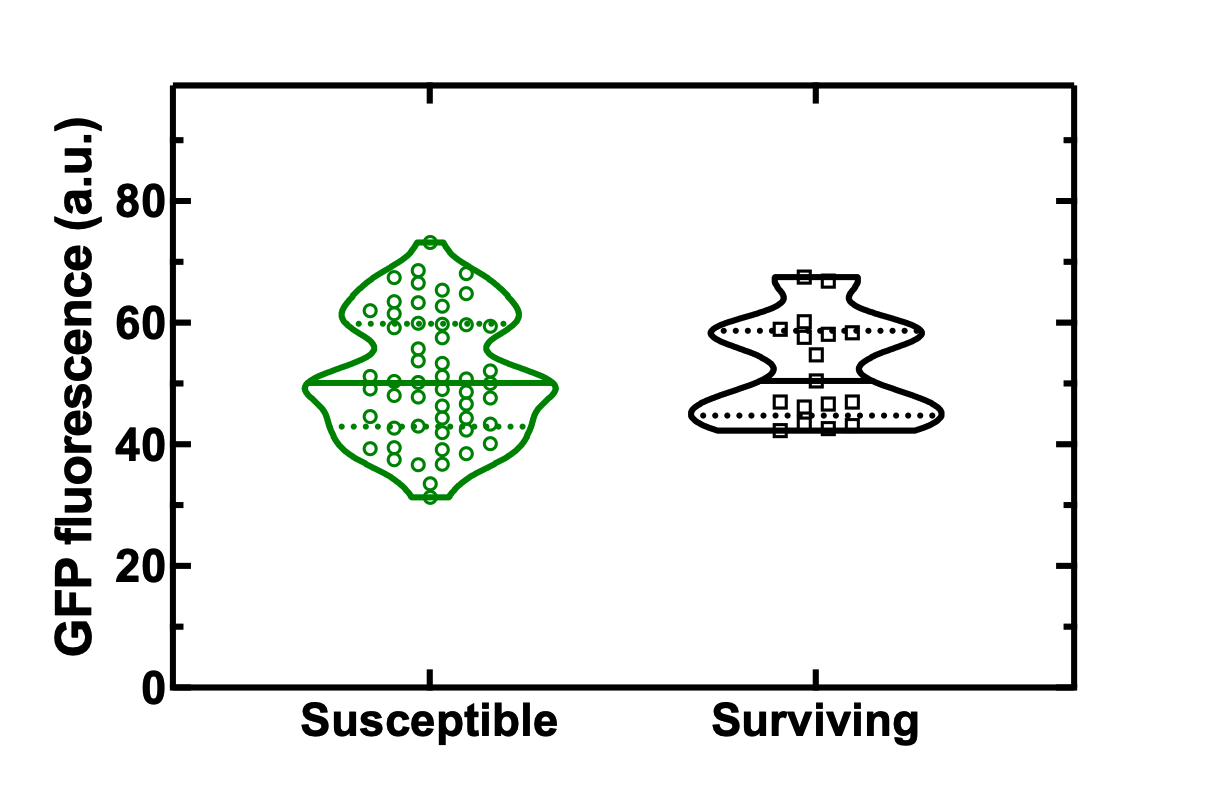

Supplement: S14 Fig — Data were obtained in biological triplicate for a total of N = 54, N = 17 bacteria that were killed or survived T4 exposure, respectively. Numerical values are provided in Data T in S1 File. GFP, green fluorescent protein; ompC, outer membrane protein C. (TIFF) [file pbio.3001406.s015.tiff]

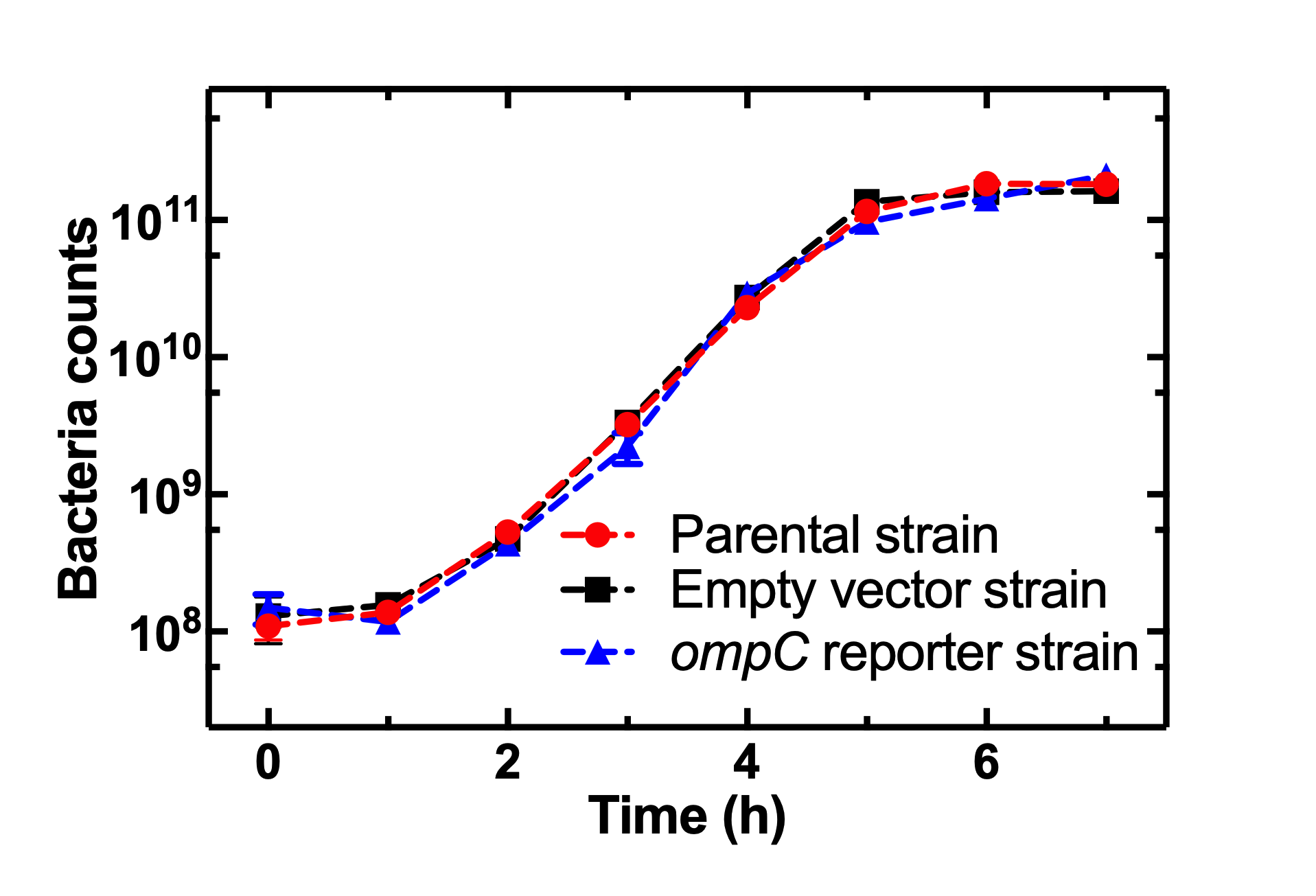

Supplement: S15 Fig — Data are the mean and standard error of the mean of biological triplicates in well-mixed 100 ml flasks. Numerical values for each replica are provided in Data U in S1 File. ompC, outer membrane protein C. (TIFF) [file pbio.3001406.s016.tiff]
